# In Silico Saturation-Mutagenesis-Based Genomic Mutation Risk Assessment for Enterovirus B

**DOI:** 10.3390/v18060645

**Published:** 2026-06-03

**Authors:** Linglin Wang, Jiajie Tang, Yongtao Jia, Xiaoxiang Tong, Xiaofeng Ying, Qin Chen, Changzheng Dong

**Affiliations:** 1 School of Public Health, Health Science Center, Ningbo University, Ningbo 315211, China; linglinwang913@163.com (L.W.); jiajietang_1998@163.com (J.T.); yongtao_jyt@163.com (Y.J.); 13023506210@163.com (X.T.); ying1647089168@163.com (X.Y.); 2 Jiaxing Center for Disease Control and Prevention, Jiaxing 314000, China; 3 Department of Public Health, Ningbo No. 2 Hospital, Ningbo 315010, China; qin_chen_php@yeah.net

**Keywords:** enterovirus B, coxsackievirus B, echovirus, saturation mutagenesis, deep mutational scanning (DMS), structural stability, receptor-binding affinity, fitness, bioinformatics, machine learning

## Abstract

Enterovirus B (EVB) is the most prevalent species of human enteroviruses, responsible for a wide range of diseases, including hand, foot, and mouth disease, viral meningitis, myocarditis, and neonatal sepsis, imposing a significant disease burden primarily on children. Coxsackievirus B (CVB1-6) and various echovirus (E) serotypes are the major serotypes of EVB. Since no antiviral drug or vaccine is available, it is important to strengthen monitoring, risk assessment, and early warning of genomic variations for EVB. CVB1, CVB3, E6, and E30 were selected as representative EVB serotypes for this study due to the availability of three-dimensional structures and their global prevalence. To evaluate the mutation effects of structural proteins on structural stability and receptor-binding affinity, computational saturation mutagenesis of EVB serotypes was performed using FoldX. Furthermore, based on data from deep mutational scanning for CVB3, a risk prediction model for EVB fitness was constructed by machine learning algorithms and applied to other EVB serotypes. Finally, we integrated three phenotypes—structural stability, receptor-binding affinity and fitness—to evaluate genomic variation risk of EVB and tracked the prevalence of high-risk mutants in natural viral sequences through molecular evolution analysis and mutation profiles. We identified the N-terminus and C-terminus of VP1 and the EF loop of VP2 as the EVB regions of highest genomic variation risk, and high-risk mutations had played significant roles in viral evolutionary history. These findings provide a framework for multi-phenotypic and multi-data approaches to viral risk assessment and offer insights to support the development of antiviral drugs and vaccines.

## 1. Introduction

Human enteroviruses belong to the *Enterovirus* genus within the *Picornaviridae* family and are classified into four species: enterovirus A (EVA), B (EVB), C (EVC), and D (EVD). Among these, EVA and EVB are the most prevalent species, responsible for a wide range of diseases, including hand, foot, and mouth disease (HFMD), viral meningitis, myocarditis, and neonatal sepsis, imposing a significant disease burden primarily on children [1,2,3,4,5,6]. EVB comprises over 60 serotypes, including coxsackievirus B (CVB1-6) and various echovirus (E) serotypes. Sun et al. [7] detected enteroviruses in samples from patients with aseptic meningitis in Zhejiang Province, China, from 2014 to 2017, identifying all serotypes as EVB. Zhang et al. [8] analyzed clinical data from 237 neonates and infants with severe enterovirus infections reported across 66 global studies from 2000 to 2020, finding that 82.7% were infected with CVB and 16.7% with echovirus. From 2018 to 2023, according to reports from the European Non-Polio Enterovirus Network (ENPEN), seven of the top ten enteroviruses were EVB serotypes [9]. Similarly, the U.S. National Enterovirus Surveillance System (NESS) reported seven EVB serotypes among the top ten enteroviruses in 2024 (https://www.cdc.gov/ness/data-vis/index.html, accessed on 2 March 2026). To date, vaccines have only been commercialized for EV71 (within EVA) and poliovirus (within EVC) [10,11]. Vaccines against other enteroviruses, such as CVA16 and CVA6, remain under development. Furthermore, no antiviral drugs specifically targeting enteroviruses have been approved for clinical use. Consequently, unlike influenza [12] or severe acute respiratory syndrome coronavirus 2 (SARS-CoV-2) infections [13], antiviral therapy is unavailable for enterovirus infections, and treatment is limited to supportive care.

Enteroviruses share a highly conserved genomic organization and viral architecture. They possess a single-stranded, positive-sense RNA genome of approximately 7.4 kilobases (kb), which encodes a single large open reading frame (ORF) [1,2,3,4,5,6]. This ORF is translated into a polyprotein that is subsequently cleaved by viral proteases into three segments (P1, P2, and P3). The P1 segment encodes the viral structural (or capsid) proteins VP1, VP2, VP3, and VP4. These four proteins assemble to form an asymmetric protomer. Five protomers constitute a pentamer, and twelve pentamers form the complete icosahedral viral capsid. VP1 to VP3 are exposed on the outer surface of the capsid, while VP4 is located on the inner surface. VP1 to VP3 are structural proteins that harbor viral antigenic epitopes and mediate interactions with host cell receptors and neutralizing antibodies [14,15,16]. CVB serotypes utilize the coxsackievirus–adenovirus receptor (CAR) for uncoating [17,18], whereas echoviruses employ the human neonatal Fc receptor (FcRn) for this purpose [14,19]. Following binding to its specific uncoating receptor on the host cell surface, the enterovirus capsid undergoes destabilization, gradual disassembly, and uncoating. This process releases the viral RNA genome into the host cell cytoplasm to initiate infection. Subsequently, the virus replicates and assembles in the host cell before being released to facilitate spread within the human population [14,15,16].

The virus possesses key phenotypes, including capsid structural stability, receptor-binding affinity, and fitness, which collectively influence viral infection, transmission, and outbreak dynamics [20,21,22,23,24]. Among these, fitness directly reflects the replicative capacity of the virus [20,21], while capsid stability and receptor-binding affinity are closely associated with viral entry and host recognition [22,23,24]. Owing to the lack of proofreading activity in the enterovirus polymerase, the viral genome exhibits a high mutation frequency. Mutations in receptor-binding sites can enhance viral binding affinity, thereby increasing infectivity and transmissibility [25,26]. Alternatively, such mutations may also alter the cellular preference for viral infection, potentially augmenting pathogenicity and contributing to severe clinical outcomes. Concurrently, mutations that affect capsid stability and fitness can modulate viral infectivity, transmissibility, and pathogenicity. Therefore, assessing the risk associated with genomic variation in human enteroviruses can aid in outbreak surveillance and early warning, as well as support the development of vaccines and antiviral therapeutics.

Deep mutational scanning (DMS) is a recently developed high-throughput and cutting-edge technique for evaluating viral mutation risks [20,27,28]. This approach involves generating vast libraries of viral mutants through mutagenesis to simulate potential mutations, followed by phenotypic characterization of key traits such as receptor-binding affinity, immune escape, and fitness. DMS has been successfully applied to study RNA viruses, including influenza virus [29,30], human immunodeficiency virus (HIV) [31,32], and enteroviruses [20,33]. Notably, during the COVID-19 pandemic, numerous DMS studies focused on receptor binding and immune escape of SARS-CoV-2, providing important support for mutant risk assessment and therapeutic antibody development. Mattenberger et al. [20] conducted a DMS study targeting fitness across the CVB3 capsid, covering 15,711 out of 16,169 (97.2%) possible non-synonymous mutations across all 851 amino acid residues. Their findings indicated that most mutations in the CVB3 capsid are deleterious to viral fitness, whereas mutations in highly variable genomic regions tend to have relatively minor effects on fitness [20,34]. Using the same CVB3 DMS platform, Álvarez-Rodríguez et al. [33] created the immune escape maps for eight human serum samples with strong anti-CVB3 immune responses. They found strong heterogeneity in immune escape among individuals, with notable variation in the most critical escape sites across different individuals. DMS requires integration of high-throughput platforms for mutant library construction, phenotypic assays, and deep sequencing, imposing substantial technical demands. Consequently, among EVB, only CVB3 has been subjected to DMS for two key phenotypes—fitness and immune escape.

Computational saturation mutagenesis is a bioinformatic method based on computational simulations for evaluating the impact of viral mutations on protein stability and protein complex binding affinity [22,23,24]. It applies molecular modeling techniques to introduce virtual mutations into viral protein structures and assesses mutational effects by analyzing changes in free energy before and after mutation [22,35]. Unlike conventional molecular modeling, computational saturation mutagenesis systematically analyzes all possible mutations at every amino acid position (each residue mutated to the other 19 residue types). This high-throughput approach shares similarities with DMS, as both can provide comprehensive and robust support for risk assessment of viral genomes. For example, Teng et al. [35] performed a study of computational saturation mutagenesis on the complete spike protein of SARS-CoV-2, covering all 18,354 possible mutations across 966 residues. Haque et al. [22] analyzed all 3705 possible mutations across 195 residues in the receptor-binding domain (RBD) of the spike protein under different pH conditions. Both studies identified multiple mutations that affect spike protein stability and its binding to the ACE2 receptor. Sharma et al. [36] conducted an analysis of computational saturation mutagenesis on the non-structural protein (NS1) of dengue virus to evaluate the impact of mutations on NS1 structural stability, thereby aiding in the identification of conserved drug targets and the screening of potential antiviral agents. Compared with DMS, computational saturation mutagenesis is not only straightforward and broadly applicable to all viruses but also yields crucial phenotypic data on protein stability and receptor-binding affinity that are vital for viral mutation risk assessment. Thus, computational saturation mutagenesis serves as a powerful complement to DMS.

In this study, we first employed an approach of computational saturation mutagenesis to assess the impact of amino acid mutations on capsid structural stability and receptor-binding affinity for representative EVB serotypes (CVB1, CVB3, E6 and E30). Subsequently, based on data of DMS for CVB3, we applied machine learning algorithms to construct a risk prediction model for EVB fitness, which was then extended to other serotypes. Finally, we integrated three phenotypes—structural stability, receptor-binding affinity, and fitness—to evaluate mutation risk of EVB and tracked the prevalence of high-risk mutants in natural viral sequences.

## 2. Materials and Methods

### 2.1. Computational Saturation Mutagenesis

CVB1, CVB3, E6, and E30 were selected as representative EVB serotypes for this study. This selection was based on two primary considerations: first, the three-dimensional (3D) structures of both the viral capsids themselves and their virus–receptor complexes have been determined (CVB1 and CVB3 utilize CAR as their uncoating receptor, while E6 and E30 utilize FcRn); second, these serotypes are among the most prevalent EVB strains circulating globally.

The 3D structural data for the capsid proteins of CVB1, CVB3, E6 and E30 (PDB IDs: 7DPF, 4GB3, 6ILP and 7C9S) and their corresponding virus–receptor complexes (PDB IDs: 7DQ1, 7VYK, 6ILM and 7C9V) were retrieved from the RCSB PDB database (https://www.rcsb.org, accessed on 21 June 2024). The corresponding amino acid sequences were obtained from the NCBI nucleotide database (https://www.ncbi.nlm.nih.gov/nucleotide, accessed on 21 June 2024). The assembly tool within PDB Tools (http://cao.labshare.cn/Tools/assembly.php, accessed on 21 June 2024) was utilized to generate pentamers from the structural protein protomers.

The impact of amino acid mutations on protein structural stability and receptor-binding affinity was estimated by calculating the changes in folding free energy (ΔΔG) and binding free energy (${\Delta \Delta G}_{binding}$) between mutant and wild-type structures, respectively. FoldX 5.0 [37], a protein engineering tool widely recognized as one of the most accurate force fields for predicting mutation-induced changes in stability and/or binding [38,39,40], was employed for all free-energy calculations. Protein structures were preprocessed using the “RepairPDB” command to correct potential structural defects. The “BuildModel” command was then applied to perform structural stability analysis on the pentameric assembly, and the “AnalyseComplex” command was used to evaluate receptor-binding affinity based on the 3D structures of the virus–receptor complexes. The standard workflow for computational saturation mutagenesis consisted of four main stages: (1) PDB file repair (performed once); (2) mutation introduction (with five replicate simulations averaged for robustness); (3) complex interaction analysis; and (4) result compilation with subsequent visualization through heatmaps and line graphs.

The viral capsid of enteroviruses is composed of asymmetric units containing VP1 to VP4. Five of these subunits assemble into a pentamer, and twelve pentamers form the complete viral capsid. Therefore, the pentamer serves as the core building block of the capsid and reflects the interactions between subunits. Its structural stability is representative of the overall viral capsid stability. The state parameters (as sourced from the original references) were set as follows: CVB1, 310 K, pH 7.4; CVB3, 298 K, pH 6.0; E6, 310 K, pH 7.4; and E30, 310 K, pH 7.4.

(1) $${\Delta \Delta G=\Delta G}_{mutant}-{\Delta G}_{wild}$$

Quantitative protein stability data can be converted into categorical ratings [35]: highly decreased (ΔΔG≥2.5 kcal/mol), decreased (ΔΔG≥0.5 kcal/mol), unchanged (−0.5<ΔΔG<0.5 kcal/mol), and enhanced (ΔΔG≤−0.5 kcal/mol) structural stability.

Similarly, the effect of mutations on receptor-binding affinity can be computed and categorized using an equivalent scaling system. The state parameters were set as follows: CVB1, 310 K, pH 7.4; CVB3, 310 K, pH 7.4; E6, 298 K, pH 7.4; and E30, 310 K, pH 7.4. The change in free energy can also be converted into categorical ratings [35]: decreased (${\Delta \Delta G}_{binding}\geq 0.1$ kcal/mol), unchanged ($-0.1<{\Delta \Delta G}_{binding}<0.1$ kcal/mol), and enhanced (${\Delta \Delta G}_{binding}\leq -0.1$ kcal/mol) binding affinity.

### 2.2. Risk Score for Fitness of CVB3

Mattenberger et al. [20] performed a DMS targeting fitness across all amino acid sites in the structural proteins of CVB3 and obtained mutational risk scores for each mutation. We acquired these risk scores from the supplementary materials of the publication. For each site, the risk scores of all 19 possible amino acid mutants were averaged, and this value was further averaged across three experimental replicates to calculate a definitive risk score of fitness for each site.

This quantitative scoring was subsequently converted into a binary classification. Given the scores’ skewed distribution—with a small number of sites exhibiting high, widely dispersed scores (indicating higher risk) and the majority showing low, tightly clustered scores (indicating lower risk)—the top 10% of sites, defined by the 90th percentile threshold, were classified as high-risk sites, while the remaining 90% were designated as low-risk sites.

### 2.3. Risk Prediction Model for Fitness

The risk prediction model for fitness was implemented using the Random Forest algorithm, facilitated by the RandomForestClassifier function from the sklearn package in Python 3.10. The target variable of the model was the classification of sites into high- or low-risk categories. The input features encompassed biofunctional, structural-biology, and molecular evolutionary characteristics of the amino acid sites. Biofunctional features included protein role (structural protein, antigenic epitope, or receptor-binding site); structural-biology features comprised secondary structure, structural location, distance from the α-carbon to the capsid center, and relative surface accessibility (RSA); and molecular evolutionary features included conservation across species and serotypes.

Antigenic epitopes and receptor-binding sites were curated from the published literature. Structural characteristics—such as secondary structure, structural location, and the distance from the α-carbon to the capsid center—were obtained from the VIPERdb database (https://viperdb.org, accessed on 27 April 2024). The RSA of residues was calculated using ESPrint 3.0 (https://espript.ibcp.fr, accessed on 6 May 2024). All available amino acid sequences of EVB structural proteins were retrieved from the NCBI nucleotide database (https://www.ncbi.nlm.nih.gov/nuccore, accessed on 17 May 2024) and the BV-BRC pathogen database (https://www.bv-brc.org, accessed on 17 May 2024). Multiple sequence alignment was performed using MAFFT 7.0 (https://mafft.cbrc.jp/alignment/software/, accessed on 17 May 2024). Subsequently, the conservation of sites across species and serotypes was computed using the entropy package in R 4.3.3.

Categorical features (e.g., structural protein type and structural location) were directly incorporated into the model. Quantitative features (e.g., information entropy and relative surface accessibility) were converted into discrete ordinal variables based on their data distribution: either into three categories (“low”, “medium”, and “high”) using quartiles or into two categories (“low” and “high”) using the 90th percentile. This transformation served two purposes: it facilitated modeling and enhanced model reliability by reducing the risk of overfitting.

Model performance was primarily evaluated using predictive accuracy metrics. Given that the number of high-risk sites (approximately 10% of total sites) was much smaller than that of low-risk sites (about 90%), the dataset exhibited significant class imbalance, which could easily lead the model to be biased toward the majority class. To address this, random over-sampling was applied to increase the number of high-risk instances, achieving a 1:1 balance with low-risk sites. Finally, the risk scores of CVB3 fitness were randomly split into training and validation sets in a 7:3 ratio for random forest modeling and evaluation. Model predictive performance was assessed using metrics including accuracy, recall, precision, and the F1-score.

### 2.4. Other Bioinformatic Methods and Computational Tools

The aligned EVB sequence files were imported into the WebLogo 3 website (https://weblogo.threeplusone.com/create.cgi, accessed on 20 December 2024) to generate sequence logos (mutational spectra) and calculate the frequency distribution of residues at each site. The molecular phylogenetic tree of EVB and the corresponding clade-defining mutations were derived from previous studies conducted in our laboratory [41]. PyMOL 3.1 was employed for the analysis of 3D structures of the capsid proteins [42]. Sequence alignment diagrams were generated using ESPrint 3.0, and heatmaps were plotted with the ggplot2 package in R 4.3.3. Correlation coefficients (Spearman’s correlation coefficients and Phi coefficient) were calculated using SPSS 23.

## 3. Results

### 3.1. Risk Scoring for Structural Stability of EVB via Computational Saturation Mutagenesis

The polyproteins P1 of CVB1, CVB3, E6 and E30 contain 848, 851, 853 and 860 amino acids, respectively. Computational saturation mutagenesis was performed to assess the structural stability of these sites. Due to missing residues in the determined 3D structures, the actual numbers of amino acid sites included in the analysis were 810, 822, 823 and 822, respectively. Spearman’s correlation coefficients of structural stability ΔΔG among EVB serotypes ranged from 0.680 to 0.759 (*p* < 0.001), indicating that their structural similarity leads to correlations in the effects of mutations on structural stability (Table S1).

**Figure 1:**
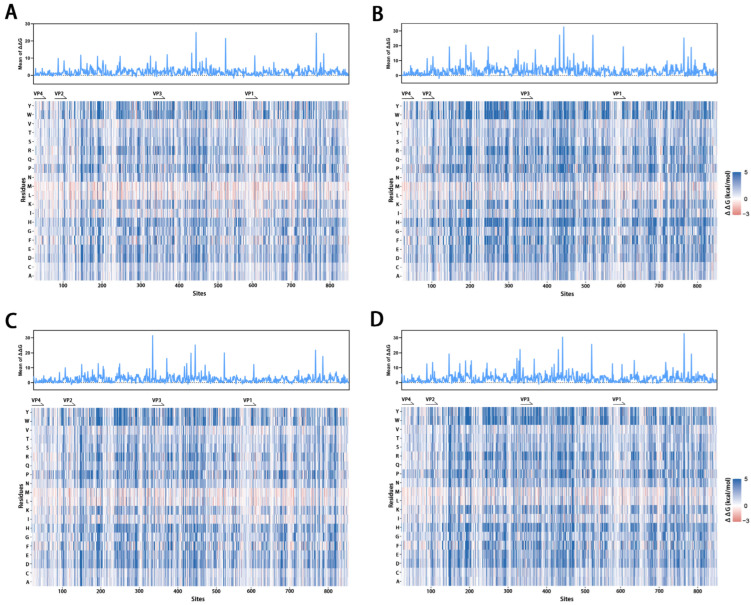
Computational saturation mutagenesis results of structural stability of EVB. The results of CVB1 (**A**), CVB3 (**B**), E6 (**C**) and E30 (**D**) are shown. The horizontal axes represent the sequence of amino acid sites on the polyprotein P1. The vertical axis of the line graphs shows the average free-energy change (kcal/mol) of each amino acid site after mutation: an increase in free energy indicates that the mutation impairs structural stability, while a decrease indicates that the mutation enhances structural stability. The vertical axis of the heatmaps shows the free-energy changes in all 19 mutations at each site: blue represents mutations that decrease structural stability, and red represents mutations that enhance structural stability. The values on the line graphs are the averages of the free-energy changes of 19 mutations at each site from the heatmaps. The heatmaps also mark the starting positions of the structural proteins VP1–VP4 on P1.

The scanning results revealed that most mutations either decreased or had no effect on structural stability; these were classified as low-risk sites. Only a very small proportion of mutations enhanced structural stability, and these were defined as high-risk sites (Table 1 and Figure 1). For example, in CVB1, 69.8% of the mutations decreased structural stability, 27.5% had no effect, and only 2.7% enhanced structural stability.

The number of high-risk sites was highly consistent across EVB serotypes, with CVB1, CVB3, E6 and E30 having 22 (2.7%), 22 (2.7%), 25 (3.0%) and 18 (2.2%) sites, respectively (Table 1 and Tables S2–S5). These high-risk sites are distributed across the structural proteins VP1–VP4. In terms of secondary structure, they are predominantly located in N-terminus, C-terminus, and loop regions (Figure S1). In the 3D structure, these sites are mainly situated at the interfaces between structural proteins, with a minority located on the outer surface (Tables S2–S5).

The distribution of stability-enhancing mutations is relatively clustered, and some mutations affect multiple serotypes. For instance, mutations at E50 and D51 in VP2 enhance the structural stability of CVB1, CVB3 and E6, while mutations at T/S64 in VP4 enhance the stability of CVB1, CVB3 and E30 (Figure S1). Moreover, the original residues at these sites are predominantly hydrophilic amino acids such as T, D, S, N and E. Mutations at these positions generally lead to enhanced structural stability (Tables S2–S5 and Figures S2–S5).

### 3.2. Risk Scoring for Receptor-Binding Affinity of EVB via Computational Saturation Mutagenesis

Similarly, due to missing residues in the determined 3D structures, the actual numbers of amino acid sites subjected to computational saturation mutagenesis for receptor-binding affinity in CVB1, CVB3, E6 and E30 were 806, 816, 840 and 822, respectively. A weak correlation (*r* = 0.317, *p* < 0.001) was observed for receptor-binding affinity ΔΔG between CVB1 and CVB3, while no correlation or only a very weak correlation was found among other EVB serotypes, suggesting that differences in receptors and receptor-binding sites result in significant differences in the effects of mutations (Table S6).

**Figure 2:**
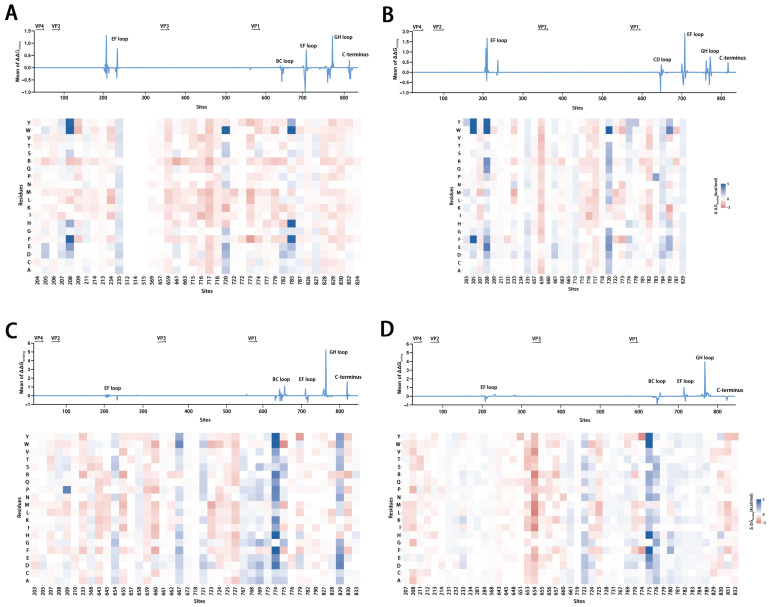
Computational saturation mutagenesis results of receptor-binding affinity of EVB. The results of CVB1 (**A**), CVB3 (**B**), E6 (**C**), and E30 (**D**) are shown.

**Figure 3:**
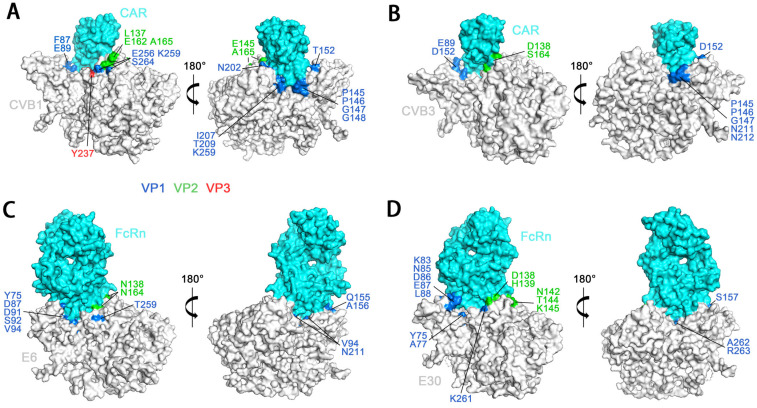
Distribution of high-risk sites on EVB. Surface views of CVB1 (**A**), CVB3 (**B**), E6 (**C**), and E30 (**D**) viral particles. The viral capsid is shown in white. Exposed high-risk sites are colored according to their capsid protein: VP1 in blue, VP2 in green, and VP3 in red (buried sites are not labeled). The receptor is displayed in cyan, with CAR as the receptor for CVB1, CVB3, and FcRn for E6 and E30. The figures illustrate the spatial distribution of high-risk sites on the viral surface and their relative positions to the receptor-binding interface for each serotype.

As expected, the vast majority of mutations did not affect receptor-binding affinity (Table 2 and Figure 2 and Figure 3). In CVB1, CVB3, E6 and E30, 26 (3.2%), 13 (1.6%), 21 (2.5%) and 21 (2.5%) sites, respectively, were defined as high-risk sites, as their mutations enhanced receptor-binding affinity. The remaining sites, where mutations had either no effect or a diminishing effect on binding affinity, were defined as low-risk sites. Among these, 9 (1.1%), 15 (1.8%), 18 (2.1%), and 21 (2.5%) sites in CVB1, CVB3, E6, and E30, respectively, were found to reduce receptor-binding affinity upon mutation (Table 2 and Tables S7–S10 and Figure S6).

In CVB1, mutations at 35 sites were found to alter receptor-binding affinity, of which 14 were direct receptor-binding sites, and 21 were neighboring sites (Table S7, Figure 2A and Figure 3A). Taking the EF loop of VP2 as an example, residues 136, 138, 139, and 140 are all receptor-binding sites. Among them, mutations at positions 136, 138, and 139 weakened binding affinity, whereas mutations at position 140 enhanced it. These results are summarized visually in a heatmap (Figure 2A). Residues VP2-136, 138, and 139 (encoded in P1 as 205, 207, and 208) predominantly show blue coloring, indicating reduced binding affinity. This effect is particularly pronounced at position 139, where the average ΔΔG reaches 1.333. In contrast, position 140 (P1-209) is mainly red, reflecting enhanced binding affinity with an average ΔΔG of −0.443.

**Figure 4:**
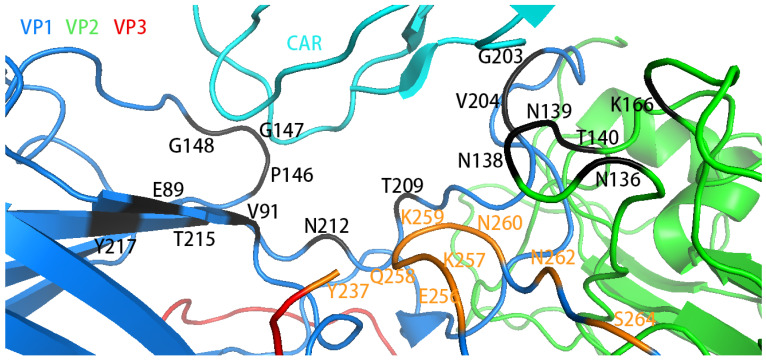
Effects of mutations in spatially adjacent sites on CVB1 receptor binding affinity. The black sites (such as T209 and N212 of VP1, N136 and N138 of VP2, etc.) are the receptor-binding sites of CVB1. The orange sites (such as E256-N260, N262, and S264 of VP1, as well as Y237 of VP3, etc.) are far from the receptor-binding sites in sequence but are spatially adjacent to these receptor-binding sites. Their mutations also affect the receptor-binding affinity of CVB1.

Among the neighboring residues, positions 135, 137 and 145 enhanced binding affinity, whereas position 142 weakened it (Figure 2A). Other adjacent sites, such as 141, 143 and 144, had no effect on receptor binding (Figure S7). Most affected neighboring sites are sequentially adjacent to receptor-binding residues. For instance, VP2-135 and VP2-137 are directly next to binding sites 136 and 138 in the amino acid sequence, while VP2-142 and VP2-145 adjoin binding sites 139 and 140 (Table S7). However, several sites—such as VP3-237 and VP1-256 to 260, 262 and 264—are located distantly from binding residues in the linear sequence but are spatially proximal in the 3D structure (Figure 4).

Mutations at only five of CVB1’s 19 CAR-binding sites (VP1-91, VP1-212, VP3-180, VP3-182 and VP3-183) were found to have no impact on receptor-binding affinity (Table S7). It is important to note that the lack of change in binding affinity at these sites actually arises from two distinct scenarios. For the vast majority of such sites, every single mutation tested had no effect on binding affinity. This category includes VP3-180, VP3-182, VP3-183, and sites distant from the binding interface (Figure 2A). In a very small number of sites, however, some individual mutations do alter binding affinity, but the average effect across all mutations at that site results in no net change. VP1-91 and 212 fall into this latter category (Figure 2A). For site VP1-91 (P1-661), specific mutations to A, D, E, G, Q, S, W or Y weakened binding affinity, while mutations to F, H, I, K, L, M, N or R enhanced it. These opposing effects cancel out, leading to an overall neutral average. Similarly, at site VP1-212 (P1-782), mutations to A, C, D, E, G, L, M, P, Q or W weakened binding affinity, whereas mutations to F, H, I, K, R, S, T or Y strengthened it. This phenomenon—where the net effect across all mutations was neutral—proved relatively rare and was observed only in a few receptor-binding sites and their neighbors.

Similarly, Figure 2B–D and Figure 3B–D and Tables S8–S10 present the computational saturation mutagenesis results for receptor-binding affinity in CVB3, E6 and E30. The analysis reveals two key patterns. First, sites affecting EVB receptor-binding are concentrated within a limited set of secondary structures. These include the BC loop, CD loop, EF loop, GH loop and C-terminus of VP1, as well as the EF loop of VP2 (Figure S6). These domains not only harbor the receptor-binding sites but also exhibit high sequence variability. Second, a limited number of conserved residues were also found to significantly affect receptor-binding affinity (Figure S6). CVB1 and CVB3, which share the common receptor—CAR, possess five conserved residues that enhance binding (VP1-E89, P145, P146, G147 and G203) and four that reduce it (VP1-V150 and T215, VP2-N139 and K166). In contrast, E6 and E30, which share the same receptor—FcRn, have only one conserved residue that enhances binding (VP1-Y75) and four that diminish it (VP1-I153, S200, G206 and V207, numbered according to E6). These residues are likely critical for receptor interaction. Among these, VP1-V150 (in CVB1 and CVB3)/I153 (in E6)/I154 (in E30) represents the sole conserved site. Across all EVB serotypes, mutations at this site resulted in reduced receptor binding affinity. Furthermore, residues 145–152 on the VP1 EF loop (numbered according to CVB3) exert nearly identical effects on receptor binding in both CVB1 and CVB3, underscoring their importance for CAR engagement. It is noteworthy that within the VP1 GH loop, while the affected sites are relatively conserved in sequence, mutations at these conserved residues (e.g., residues 208–220) can have divergent effects on binding affinity across serotypes. This suggests the potential involvement of epistatic effects, where sequence differences between serotypes modulate the phenotypic outcome of mutations.

### 3.3. Risk Scoring of EVB Fitness Derived from Deep Mutational Scanning and a Random Forest Model

Based on the DMS study of CVB3 fitness, it was found that most mutations in the CVB3 P1 are deleterious to viral fitness, and the fitness score data exhibit a skewed distribution (Figure S8). We defined the top 10% of the 821 amino acid sites in the CVB3 P1 (with fitness scores missing for 30 sites) as high-risk sites and the remaining 90% as low-risk sites, resulting in the identification of 82 high-risk fitness sites (Table 3 and Table S11) and 739 low-risk sites.

Since enterovirus DMS data for fitness is only available for CVB3, we employed a machine learning approach—the Random Forest algorithm—to construct a predictive model for assessing fitness risk across other EVB serotypes. The model treated risk sites (categorized as high-risk and low-risk) as the target variable and incorporated multiple bioinformatic features as predictor variables, including structural protein identity, Shannon entropy, secondary structure, structural location, distance of the α-carbon to the viral center, relative solvent accessibility, epitope residues, and receptor-binding sites (Table S12).

To address the severe class imbalance, where high-risk sites constituted only 10% of the total compared to 90% for low-risk sites—a skew that could bias the model toward ignoring the minority class—we applied an over-sampling re-sampling technique. This involved randomly re-sampling the high-risk sites with replacement to increase their representation. After re-sampling, the fitness dataset contained 1478 sites, with an equal number of 739 high-risk and 739 low-risk sites. Thus, the number of low-risk sites remained unchanged, while the high-risk sites increased from 82 to 739, representing an average expansion of approximately 8-fold per original high-risk site.

The resampled dataset was then split into training and testing sets in a 7:3 ratio. On the training set, the model achieved an overall predictive accuracy of 97.2%. For high-risk sites, it attained a recall of 100%, precision of 94.7%, and an F1-score of 97.3%. For low-risk sites, the recall and precision were 94.4% and 100%, respectively, yielding an F1-score of 97.1% (Table S13). On the test set, the model maintained high performance, with an overall accuracy of 96.4%. High-risk sites showed 100% recall, 93.2% precision, and a 96.5% F1-score, while low-risk sites achieved 92.9% recall, 100% precision, and a 96.3% F1-score. These results demonstrate that the model possesses high predictive accuracy and is suitable for predicting fitness risk in EVB serotypes. The Phi correlation coefficients of fitness ΔΔG among EVB serotypes ranged from 0.242 to 0.668 (*p* < 0.001), indicating that their phylogenetic relatedness leads to correlations in the effects of mutations on fitness (Table S14).

Based on the predictive model, 97, 60 and 115 high-risk fitness sites were identified for CVB1, E6 and E30, respectively (Table 3 and Figure S9). These high-risk sites are predominantly located on VP1-VP3. The N-terminus of VP1 harbors a high concentration of these sites, with one cluster in the 9–27 (in VP1) region and another in the 41–66 (in VP1) region (numbered according to CVB3). Among these, A15 and H52 are conserved high-risk sites across all four serotypes, with several others conserved across three. The C-terminus of VP1 also contains a significant cluster of high-risk sites. Sites 257, 264, 271 and 273 are high-risk in all four serotypes, although they are not conserved residues. The EF loop of VP2 is another hotspot, with sites 143 and 153 identified as high-risk across all serotypes. Similarly, the N-terminus and EF loop of VP3 are enriched with high-risk sites, including sites 35, 59, 143 and 144, which are high-risk in all serotypes. VP4 contains only 2–3 high-risk sites; notably, T47 is a high-risk site in three serotypes. In summary, the primary regions concentrating fitness high-risk sites across EVB serotypes are the N- and C-terminus of VP1, the EF loop of VP2, and the N-terminus and EF loop of VP3.

### 3.4. Comprehensive Risk Score of EVB

On the basis of risk assessment for individual phenotypes (structural stability, receptor-binding affinity, and fitness) in EVB, further comprehensive risk assessment can be performed by integrating all three phenotypes. For example, a mutation at a high-risk site that simultaneously enhances receptor-binding affinity and fitness, and even enhances structural stability, would confer a multi-phenotypic advantage that is highly favorable for viral survival. Conversely, a mutation at a site that highly decreases structural stability, even if it enhances receptor-binding affinity or fitness, may not confer a realistic survival advantage. Based on this hypothesis, Figures S10–S13 summarize high-risk sites across the three phenotypes in EVB, enabling comprehensive assessment of mutational risk.

It can be clearly observed from the figures that mutations at some high-risk sites affecting single or even multiple phenotypes actually highly decrease structural stability. For example, the VP2-207 mutation enhances receptor-binding affinity and fitness of CVB3, but it simultaneously highly decreases structural stability, resulting in a relatively low probability of actual mutation occurrence. The VP2-233 mutation simultaneously enhances structural stability, receptor-binding affinity, and fitness of CVB3, conferring a significant phenotypic advantage. Among the results of computational saturation mutagenesis, there are also a few sites (e.g., VP3-181 and 232) where mutations simultaneously enhance both structural stability and fitness of CVB3, and a few sites (e.g., VP2-138) where mutations simultaneously enhance both receptor-binding affinity and fitness of CVB3 without decreasing capsid structural stability, also exhibiting relatively prominent phenotypic advantages. Similarly, mutations at sites such as VP2-140/162/165 in CVB1, VP1-259 and VP2-141 in E6, and VP2-138/139/142/143 in E30, although enhancing receptor-binding affinity and fitness, all highly decrease structural stability, rendering these sites less prone to mutation in nature. Mutations at VP1-86 and VP2-144 in E30 simultaneously enhance all three phenotypes. Mutations at sites such as VP1-256 and 264 in CVB1, VP1-259 in E6, and VP1-261 and 263 in E30 enhance receptor-binding affinity and fitness while maintaining relatively stable capsid structure (without significantly decreasing structural stability), conferring significant advantages. In addition, many high-risk sites, when mutated, enhance only a single phenotype (structural stability, receptor-binding affinity, or fitness) without significantly affecting the other phenotypes.

Comparing the structural stability risk sites and mutation profiles of EVB (Figures S10–S17), it can be observed that the vast majority of high-frequency mutation sites are not extremely-low-risk sites for structural stability, despite extremely-low-risk sites accounting for approximately 30–40% of all sites (Table 1). In other words, none of the mutations in the natural sequences significantly decrease structural stability. For example, high-frequency mutation sites such as VP1-18/64/84/85, VP2-13/45/144/151, and VP3-35/58/78 in CVB3 are not extremely-low-risk sites; similarly, high-frequency mutation sites such as VP2-144/151/153 in CVB1, VP2-43/45/136 in E6, and VP2-37/74/156 in E30 are also not extremely-low-risk sites. These pieces of evidence validate our hypothesis: mutations at sites that highly decrease structural stability do not confer a realistic survival advantage, and extremely-low-risk sites for structural stability can serve as restrictive conditions for viral phenotypic evolution.

**Figure 5:**
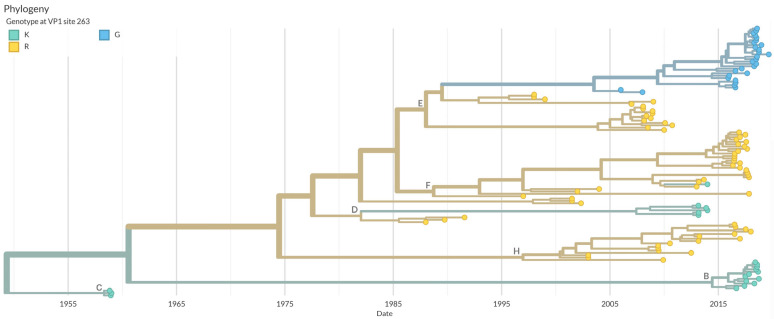
Multiple mutations have occurred at VP1-263, a high-risk site in E30. Based on whole genome sequences, the E30 molecular phylogenetic tree contains major evolutionary clades B, C, D, E, F, and H. Multiple reverse mutations were observed at the high-risk site VP1-263, with green indicating the amino acid residue K, yellow indicating G, and blue indicating R.

Additionally, it can be observed that some high-risk sites have already undergone high-frequency mutations. For example, VP2-51 and 234 (high-risk sites for structural stability in CVB1), VP2-162 and 165 (high-risk sites for both receptor-binding affinity and fitness in CVB1); VP2-164 (a high-risk site for all three phenotypes in CVB3), VP2-45, 144, 151 and 156 (high-risk sites for fitness in CVB3); VP3-93 (a high-risk site for structural stability in E6), VP1-11, 18 and 19 (high-risk sites for fitness in E6); VP2-45 (a high-risk site for both structural stability and fitness in E30) and VP1-263 (a high-risk site for both receptor-binding affinity and fitness in E30). Notably, multiple independent mutations occurring at some high-risk sites have become hallmark mutations of different evolutionary clades, such as VP1-263 and VP2-45 in E30 (Figure 5 and Figure S18). These findings suggest that the prevalence of mutations at high-risk sites requires close monitoring, and the effects of these mutations warrant further experimental validation.

## 4. Discussion

Bioinformatics has been widely applied in the field of viral mutation risk assessment. For instance, Smith et al. [43] developed antigenic cartography based on hemagglutination inhibition (HI) assay data, which reduces multi-dimensional antigenic data via multi-dimensional scaling to construct two-dimensional antigenic maps for influenza viruses. This method effectively predicts antigenic shifts and has been adopted by the WHO to inform influenza vaccine recommendations. Han et al. [44] employed multiple machine learning approaches to construct antigenicity prediction models for the Victoria and Yamagata clades of influenza B virus. Their work systematically elucidated the antigenic evolution patterns of these clades and revealed potential mechanisms behind the disappearance of the Yamagata clade. Li et al. [45] utilized a deep-learning algorithm (a three-dimensional convolutional neural network) to assess the fitness of SARS-CoV-2 variants based on genomic features.

Computational saturation mutagenesis follows a logic similar to DMS by virtually substituting each amino acid residue with the other 19 possible residues. Based on the 3D structure of the virus, computational saturation mutagenesis calculates the difference in free energy between mutant and wild-type variants, estimates the impact of mutational free-energy changes on protein structural stability or receptor-binding affinity, and thereby enables risk assessment of viral mutations. As a structure-based computational approach, computational saturation mutagenesis does not rely on serological or other experimental data, yet it captures the relationship between sequence variation and certain phenotypes—such as structural stability, receptor-binding affinity and antibody escape—more directly than sequence-only analyses. Studies by Haque et al., Sun et al., and Teng et al. [22,35,46] have applied computational saturation mutagenesis to SARS-CoV-2, and identified numerous mutations that affect protein structural stability and receptor-binding affinity. Sharma et al. [36] performed computational saturation mutagenesis on the NS1 protein of dengue virus and found that most mutations destabilize NS1, with these destabilizing mutations predominantly located in conserved regions, whereas the few stabilizing mutations tend to occur in high-frequency mutation sites. For enteroviruses, comprehensive serological data are often lacking. However, the 3D structures of several serotypes—both of the viral capsid alone and in complex with their receptors—have been determined. Therefore, computational saturation mutagenesis presents an ideal computational strategy for risk assessment in enteroviruses.

Saturation mutagenesis of EVB structural stability revealed that approximately 70–80% of sites exhibit reduced stability upon mutation, with 30–50% of sites showing severely destabilizing effects. These destabilizing mutations are harmful to viral capsid integrity and are therefore classified as low-risk sites. In contrast, only about 2–3% of mutations enhance structural stability; these are defined as high-risk sites. This pattern suggests that enteroviruses have evolved a highly optimized capsid architecture. The remaining 20–30% of mutations do not change stability and are considered neutral from a structural perspective, thus also categorized as low-risk sites; mutations at these positions are not constrained by structural stability phenotype. Sites that enhance stability are predominantly located at interfaces in the three-dimensional structure (Tables S2–S5) and within flexible regions such as the N-terminus, C-terminus and loops in secondary structure (Figure S1).

Interestingly, this study found that across CVB1, CVB3, E6 and E30, mutations to leucine (Leu, L) or methionine (Met, M) generally enhanced structural stability as predicted by computational saturation mutagenesis, and this effect was consistent across different serotypes (Figure 1). Both leucine and methionine are hydrophobic amino acids, and their stabilizing effects can be explained by classical principles of protein folding. When the mutation site is located in the protein core and the original amino acid has weak hydrophobicity or a small side chain, mutation to leucine (bulky and highly hydrophobic) or methionine (flexible long chain) confers two benefits: first, a stronger hydrophobic driving force—the hydrophobic effect is the primary driving force for protein folding, and burying nonpolar side chains in the protein interior significantly reduces the free energy of the system [47]; second, better space filling—filling internal cavities in proteins has been established as an important mechanism of stabilizing mutations, and larger side chains can more effectively fill voids in the core, enhancing van der Waals interactions [48]. Conversely, when the mutation site is on the protein surface, mutation to a hydrophobic amino acid results in exposure of the hydrophobic side chain to the aqueous solvent, incurring an unfavorable hydrophobic solvation effect. These mechanisms are highly consistent with the structural characteristics of enterovirus capsid proteins. VP1, VP2, and VP3 all adopt β-barrel folds, with their core regions composed of hydrophobic amino acids that are largely buried, while VP4 is located entirely on the inner surface of the capsid. Therefore, the observed phenomenon that leucine and methionine mutations generally enhance structural stability across EVB serotypes can be reasonably explained. However, this is only a theoretical analysis; the actual effects of mutations are also related to the complex interactions within residue networks, and thus further experimental validation is required.

Saturation mutagenesis of EVB receptor-binding affinity showed that only about 3–5% of mutations alter binding affinity (either enhancing or decreasing). These include known receptor-binding sites and adjacent sites, with neighboring positions accounting for 45–80% of the affected sites (Tables S7–S10). Most adjacent sites are proximate in the amino acid sequence, while a minority are spatially adjacent despite being distant in the linear sequence. Notably, even among structurally defined receptor-binding sites (Some studies define receptor-binding sites based on a distance of less than 4 Å between viral and receptor residues), a subset showed no significant effect upon mutation, suggesting they may not play critical roles in binding. Only 10–30 sites enhance binding affinity upon mutation, benefiting the virus and thus defined as high-risk sites; all others are classified as low-risk sites. Sites that influence EVB receptor-binding affinity are concentrated in secondary structural regions including the BC loop, CD loop, EF loop, GH loop and C-terminus of VP1, as well as the EF loop of VP2 (Figure S6). These regions not only harbor receptor-binding sites but also exhibit high variability of sequence. The scanning further identified conserved residues (VP1-E89, P145, P146, G147 and G203) that enhance CAR binding in both CVB1 and CVB3. Moreover, E6 and E30, which both bind FcRn, share only one conserved binding-enhancing residue (VP1-Y75). Compared with CVB, it indicates that echoviruses, which show greater sequence and structural divergence among serotypes, possess more diverse high-risk sites. VP1–V150 (in CVB1 and CVB3)/I153 (in E6)/I154 (in E30) is the sole conserved site affecting receptor-binding affinity across all EVB serotypes; mutations at this position universally reduce binding affinity, underscoring its functional importance. Similarly, the 145–152 (in VP1) segment is critical for CAR binding in CVB1 and CVB3. Together, these findings strongly support that computational saturation mutagenesis provides a valuable complement to structural biology and offer predictive insights into the phenotypic impact of mutations beyond static three-dimensional models.

The DMS data on CVB3 fitness provide a viable approach for assessing fitness risk across EVB serotypes. Utilizing biofunctional, structural, and molecular evolutionary features of each site, a random forest prediction model achieved high accuracy in forecasting CVB3 fitness, with overall accuracy exceeding 96% on both training and test datasets, and the lowest individual evaluation metric reaching 92.9%. When extended to other EVB serotypes, high-risk sites were found to cluster in specific regions, including the N-terminus and C-terminus of VP1, the EF loop of VP2, and the N-terminus and EF loop of VP3. Álvarez-Rodríguez et al. [33] conducted a DMS study on CVB3 immune escape, which initially offered an opportunity to assess immune escape risk for EVB. Unfortunately, the eight serum samples they selected were obtained from healthy donors with strong anti-CVB3 immune responses rather than from convalescent patients. This resulted in generally weak neutralization and limited mutation-escape maps, alongside pronounced inter-individual heterogeneity in DMS outcomes, making it difficult to identify common escape sites. Consequently, this dataset was not employed in our study.

Viral survival, transmission, and evolution are shaped by multiple phenotypes simultaneously [27,28,49,50,51,52]. Mutations that enhance immune escape may decrease structural stability or receptor-binding affinity; thus, an advantage in a single phenotype does not necessarily confer a survival or competitive advantage [27,50,51,52]. Conversely, mutations that confer benefits across multiple phenotypes are likely to possess a stronger competitive advantage. This logic has been validated in studies on SARS-CoV-2: a large number of DMS studies have shown that effective immune escape mutations typically do not significantly disrupt the two phenotypes of viral protein folding and receptor-binding affinity. Based on the same concept, this study further established a comprehensive risk scoring model for the three phenotypes (structural stability, receptor-binding affinity, and fitness) of EVB, building upon the single-phenotype risk assessment [27,50,51,52].

The comprehensive risk assessment results clearly demonstrate that structural stability serves as a key constraint condition for viral phenotypic evolution. Bloom et al. [53], in their study on protein stability and evolvability, proposed a critical mechanism: additional stability itself is neutral with respect to function, but it is a prerequisite for proteins to tolerate beneficial mutations. Without sufficient stability margin, even mutations conferring functional advantages will be eliminated due to disruption of structural integrity. The present study validates this mechanism in EVB. Mutations at sites that highly decrease structural stability, even if they enhance receptor-binding affinity or fitness, are rarely observed in natural populations. For example, the VP2-207 mutation in CVB3 enhances receptor-binding affinity and fitness, but because it highly decreases structural stability, the probability of its actual occurrence is extremely low (Figure S11). Similarly, sites such as VP2-140 in CVB1, VP2-141 in E6, and VP2-138 in E30 show the same pattern (Figures S10, S12 and S13). Sites that highly decrease structural stability are mainly distributed at the interfaces between asymmetric units, which is highly similar to the conclusion of a study comparing DMS results of fitness between EVA and EVB [54]. In their study, the capsid assembly interface was identified as a common evolutionary constraint region for both EVA and EVB.

Notably, this study also identified a set of “all-beneficial” sites that simultaneously enhance all three phenotypes (e.g., VP2-233 in CVB3 and VP1-86 and VP2-144 in E30), as well as a set of “beneficial” sites that enhance receptor-binding affinity and fitness while maintaining structural stability (e.g., VP1-256 and 264 in CVB1, VP1-259 in E6, and VP1-261 and 263 in E30) (Figures S10–S13). The existence of these “beneficial” sites suggests that viruses can achieve multi-phenotypic synergistic optimization without sacrificing structural integrity. The biophysical model proposed by Echave and Wilke [55] indicates that the slowest-evolving sites in proteins tend to be those that most strongly affect structural stability, whereas regions that are more robust (more tolerant) to mutations evolve faster. In this study, these “beneficial” sites are precisely located in structural regions with high tolerance to mutations (the N-terminus and C-terminus of VP1 and the EF loop of VP2 are “hotspot” regions for high-risk sites across EVB serotypes, exhibiting the highest mutational risk), allowing them to explore functional gains while maintaining or even enhancing stability. This observation is highly consistent with theoretical expectations.

Analysis of the actual prevalence of these predicted high-risk sites in nature revealed that some high-risk sites have already undergone high-frequency mutations in natural populations, and mutations at some of these sites have even become hallmark mutations of distinct evolutionary clades. For example, sites such as VP2-164 in CVB3, VP1-263 and VP2-45 in E30, not only possess theoretical multi-phenotypic advantages but are also favored by natural selection in the real world. We speculate that the sequence backgrounds of these sites may have acquired sufficient structural stability “margin” through prior neutral mutations, thus providing a tolerance window for subsequent gain-of-function mutations.

This study also has several limitations. First, computational saturation mutagenesis relies on the availability of 3D viral structures; the lack of a three-dimensional structure (e.g., the absence of a virus-antibody complex structure for EVB) restricts the feasibility of such studies. Second, FoldX, as a protein energy calculation tool, has an accuracy that depends on the atomic resolution of the input structure; low resolution or structural errors may be amplified in the calculation of mutation free energy. Furthermore, FoldX has limited capacity for fine characterization of local structural details, which is similar to high-throughput experimental techniques such as DMS—both prioritize coverage breadth over atomic-level precision. In addition, to reduce complexity, this study focused on evaluating the overall mutational effect at each site, without dissecting the impact of specific amino acid substitutions. In reality, even high-risk sites may occasionally harbor low-risk mutations, though, from an evolutionary perspective, such positions represent greater risk. The study also did not account for epistasis, i.e., the non-additive interactions among multiple mutations that may lead to synergistic, suppressive, or compensatory effects. Although this simplifies the model and facilitates high-throughput analysis, it may overestimate or underestimate the actual risk of certain mutation combinations. These limitations suggest that future studies should integrate molecular dynamics (MD) simulations and double/multiple-mutation experiments to complement the current approach. As a bioinformatics method, computational saturation mutagenesis provides candidate mutations for experimental validation, which requires further verification.

## 5. Conclusions

In summary, our work integrated computational saturation mutagenesis with DMS data to comprehensively assess variation risk in EVB. The results identify the N-terminus and C-terminus of VP1 and the EF loop of VP2 as the regions of highest variational risk. These findings provide a framework for multi-phenotypic and multi-data approaches to viral risk assessment and offer insights to support the development of antiviral drugs and vaccines.

## Figures and Tables

**Table 1:** Risk scores for structural stability of EVB sites [*n* (%)].

Serotype	Risk Score	Structural Stability Grade	VP4	VP2	VP3	VP1	Total
CVB1	High	Enhanced	1 (1.8)	7 (2.8)	5 (2.1)	9 (3.3)	22 (2.7)
	Low	Unchanged	26 (48.2)	70 (27.8)	55 (23.2)	72 (27.0)	223 (27.5)
	Low	Decreased	22 (40.7)	89 (35.3)	90 (38.0)	114 (42.7)	315 (38.9)
	Extremely-low	Highly decreased	5 (9.3)	86 (34.1)	87 (36.7)	72 (27.0)	250 (30.9)
CVB3	High	Enhanced	1 (1.8)	5 (1.9)	8 (3.3)	8 (2.9)	22 (2.7)
	Low	Unchanged	24 (43.6)	57 (22.3)	39 (16.4)	60 (22.0)	180 (21.9)
	Low	Decreased	21 (38.2)	76 (29.7)	59 (24.8)	76 (27.8)	232 (28.2)
	Extremely-low	Highly decreased	9 (16.4)	118 (46.1)	132 (55.5)	129 (42.3)	388 (47.2)
E6	High	Enhanced	2 (3.4)	6 (2.4)	6 (2.5)	11 (4.0)	25 (3.0)
	Low	Unchanged	25 (43.1)	65 (25.8)	47 (19.8)	70 (25.4)	207 (25.2)
	Low	Decreased	23 (39.7)	84 (33.3)	96 (40.3)	114 (41.5)	317 (38.5)
	Extremely-low	Highly decreased	8 (13.8)	97 (38.5)	89 (37.4)	80 (29.1)	274 (33.3)
E30	High	Enhanced	1 (1.7)	6 (2.4)	4 (1.7)	7 (2.5)	18 (2.2)
	Low	Unchanged	24 (42.1)	57 (22.7)	40 (16.8)	61 (22.1)	182 (22.0)
	Low	Decreased	23 (40.4)	79 (31.5)	69 (29.0)	95 (34.4)	266 (32.4)
	Extremely-low	Highly decreased	9 (15.8)	109 (43.4)	125 (52.5)	113 (41.0)	356 (43.4)

**Table 2:** Risk scores for receptor-binding affinity of EVB sites [*n* (%)].

Serotype	Risk Score	Binding Affinity Grade	VP4	VP2	VP3	VP1	Total
CVB1	High	Enhanced	0 (0.0)	6 (2.4)	1 (0.4)	19 (7.1)	26 (3.2)
	Low	Unchanged	50 (100.0)	241 (95.6)	236 (99.6)	244 (91.4)	771 (95.7)
	Low	Decreased	0 (0.0)	5 (2.0)	0 (0.0)	4 (1.5)	9 (1.1)
CVB3	High	Enhanced	0 (0.0)	2 (0.7)	0 (0.0)	11 (4.1)	13 (1.6)
	Low	Unchanged	57 (100.0)	248 (97.3)	237 (100.0)	246 (92.1)	788 (96.6)
	Low	Decreased	0 (0.0)	5 (2.0)	0 (0.0)	10 (3.8)	15 (1.8)
E6	High	Enhanced	0 (0.0)	4 (1.6)	0 (0.0)	17 (5.9)	21 (2.5)
	Low	Unchanged	61 (100.0)	245 (97.2)	237 (99.6)	258 (89.3)	801 (95.4)
	Low	Decreased	0 (0.0)	3 (1.2)	1 (0.4)	14 (4.8)	18 (2.1)
E30	High	Enhanced	0 (0.0)	6 (2.4)	0 (0.0)	15 (5.5)	21 (2.5)
	Low	Unchanged	57 (100.0)	239 (95.2)	238 (100.0)	246 (89.0)	780 (95.0)
	Low	Decreased	0 (0.0)	6 (2.4)	0 (0.0)	15 (5.5)	21 (2.5)

**Table 3:** High-risk sites for fitness of EVB [*n* (%)].

Serotype	VP4	VP2	VP3	VP1	Total
CVB1	3 (3.1)	22 (22.7)	21 (21.6)	51 (52.6)	97 (100.0)
CVB3	2 (2.4)	21 (25.6)	19 (23.2)	40 (48.8)	82 (100.0)
E6	2 (3.3)	13 (21.7)	11 (18.3)	34 (56.7)	60 (100.0)
E30	2 (1.7)	29 (25.2)	24 (20.9)	60 (52.2)	115 (100.0)

## Data Availability

The data presented in this study are available on request from the corresponding author.

## Supplementary Materials

The following supporting information can be downloaded at https://www.mdpi.com/article/10.3390/v18060645/s1. Table S1: Correlation of structural stability ΔΔG among EVB serotypes; Table S2: Sites enhancing the structural stability of CVB1 after mutation; Table S3: Sites enhancing the structural stability of CVB3 after mutation; Table S4: Sites enhancing the structural stability of E6 after mutation; Table S5: Sites enhancing the structural stability of E30 after mutation; Table S6: Correlation of receptor-binding ΔΔG among EVB serotypes; Table S7: Sites affecting CVB1 receptor-binding affinity after mutation; Table S8: Sites affecting CVB3 receptor-binding affinity after mutation; Table S9: Sites affecting E6 receptor-binding affinity after mutation; Table S10: Sites affecting E30 receptor-binding affinity after mutation; Table S11: High-risk sites for fitness of CVB3; Table S12: Bioinformatics characteristics of CVB3 [n (%)]; Table S13: Prediction results of the EVB fitness risk prediction model; Table S14: Correlation of fitness risk scores among EVB serotypes; Figure S1: Sequence alignment of high-risk sites for structural stability of EVB; Figure S2: Heatmap of sites enhancing the structural stability of CVB1 after mutation; Figure S3: Heatmap of sites enhancing the structural stability of E6 after mutation; Figure S4: Heatmap of sites enhancing the structural stability of E6 after mutation; Figure S5: Heatmap of sites enhancing the structural stability of E30 after mutation; Figure S6: Sequence alignment of high-risk sites for receptor-binding affinity of EVB; Figure S7: Computational saturation mutagenesis on receptor-binding affinity of partial sites in CVB1; Figure S8: Frequency distribution of CVB3 fitness scores; Figure S9: Sequence alignment of high-risk sites for fitness of EVB; Figure S10: Sequence alignment of risk sites for CVB1; Figure S11: Sequence alignment of risk sites for CVB3; Figure S12: Sequence alignment of risk sites for E6; Figure S13: Sequence alignment of risk sites for E30; Figure S14: The mutation profile of CVB1; Figure S15: The mutation profile of CVB3; Figure S16: The mutation profile of E6; Figure S17: The mutation profile of E30; and Figure S18: Multiple mutations have occurred at VP2-45, a high-risk site in E30.

## Abbreviations

The following abbreviations are used in this manuscript:

EVA	Enterovirus A
EVB	Enterovirus B
EVC	Enterovirus C
EVD	Enterovirus D
HFMD	Hand, foot, and mouth disease
CV	Coxsackievirus
E	Echovirus
ENPEN	European Non-Polio Enterovirus Network
NESS	U.S. National Enterovirus Surveillance System
SARS-CoV-2	Severe acute respiratory syndrome coronavirus 2
kb	Kilobases
ORF	Open reading frame
VP	Viral protein
CAR	Coxsackievirus and adenovirus receptor
FcRn	Human neonatal Fc receptor
DMS	Deep mutational scanning
HIV	Human immunodeficiency virus
RBD	Receptor-binding domain
NS1	Non-structural protein
3D	Three-dimensional
RSA	Relative surface accessibility
HI	Hemagglutination inhibition

## References

[1] Chen X.P., Sun S.Z., Guo J.Y., Li J.J., Xie Z.D. (2016). Complete Genome Sequence Analysis of Echovirus 18 Associated with Aseptic Meningitis in Hebei Province, China, in 2015. Genome Announc..

[2] Ji T., Guo Y., Huang W., Shi Y., Xu Y., Tong W., Yao W., Tan Z., Zeng H., Ma J. (2018). The emerging sub-genotype C2 of CoxsackievirusA10 Associated with Hand, Foot and Mouth Disease extensively circulating in mainland of China. Sci. Rep..

[3] Krumbholz A., Egerer R., Braun H., Schmidtke M., Rimek D., Kroh C., Hennig B., Groth M., Sauerbrei A., Zell R. (2016). Analysis of an echovirus 18 outbreak in Thuringia, Germany: Insights into the molecular epidemiology and evolution of several enterovirus species B members. Med. Microbiol. Immunol..

[4] Song Y., Zhang Y., Han Z., Xu W., Xiao J., Wang X., Wang J., Yang J., Yu Q., Yu D. (2020). Genetic recombination in fast-spreading coxsackievirus A6 variants: A potential role in evolution and pathogenicity. Virus Evol..

[5] Zhang Y., Tan X., Cui A., Mao N., Xu S., Zhu Z., Zhou J., Shi J., Zhao Y., Wang X. (2013). Complete genome analysis of the C4 subgenotype strains of enterovirus 71: Predominant recombination C4 viruses persistently circulating in China for 14 years. PLoS ONE.

[6] Zhang Y., Zhu Z., Yang W., Ren J., Tan X., Wang Y., Mao N., Xu S., Zhu S., Cui A. (2010). An emerging recombinant human enterovirus 71 responsible for the 2008 outbreak of hand foot and mouth disease in Fuyang city of China. Virol. J..

[7] Sun Y., Miao Z., Yan J., Gong L., Chen Y., Chen Y., Mao H., Zhang Y. (2019). Sero-molecular epidemiology of enterovirus-associated encephalitis in Zhejiang Province, China, from 2014 to 2017. Int. J. Infect. Dis..

[8] Zhang M., Wang H., Tang J., He Y., Xiong T., Li W., Qu Y., Mu D. (2021). Clinical characteristics of severe neonatal enterovirus infection: A systematic review. BMC Pediatr..

[9] de Schrijver S., Vanhulle E., Ingenbleek A., Alexakis L., Johannesen C.K., Broberg E.K., Harvala H., Fischer T.K., Benschop K.S.M. (2025). Epidemiological and Clinical Insights into Enterovirus Circulation in Europe, 2018-2023: A Multicenter Retrospective Surveillance Study. J. Infect. Dis..

[10] Fang C.Y., Liu C.C. (2018). Recent development of enterovirus A vaccine candidates for the prevention of hand, foot, and mouth disease. Expert Rev. Vaccines.

[11] He X., Zhang M., Zhao C., Zheng P., Zhang X., Xu J. (2021). From Monovalent to Multivalent Vaccines, the Exploration for Potential Preventive Strategies Against Hand, Foot, and Mouth Disease (HFMD). Virol. Sin..

[12] Świerczyńska M., Mirowska-Guzel D.M., Pindelska E. (2022). Antiviral Drugs in Influenza. Int. J. Environ. Res. Public Health.

[13] Ratan Y., Rajput A., Jain V., Mishra D.K., Gautam R.K., Pareek A. (2023). Promising Repurposed Antiviral Molecules to Combat SARS-CoV-2: A Review. Curr. Pharm. Biotechnol..

[14] Wang K., Zhu L., Sun Y., Li M., Zhao X., Cui L., Zhang L., Gao G.F., Zhai W., Zhu F. (2020). Structures of Echovirus 30 in complex with its receptors inform a rational prediction for enterovirus receptor usage. Nat. Commun..

[15] Zhao Y., Zhou D., Ni T., Karia D., Kotecha A., Wang X., Rao Z., Jones E.Y., Fry E.E., Ren J. (2020). Hand-foot-and-mouth disease virus receptor KREMEN1 binds the canyon of Coxsackie Virus A10. Nat. Commun..

[16] Zhou D., Zhao Y., Kotecha A., Fry E.E., Kelly J.T., Wang X., Rao Z., Rowlands D.J., Ren J., Stuart D.I. (2019). Unexpected mode of engagement between enterovirus 71 and its receptor SCARB2. Nat. Microbiol..

[17] Organtini L.J., Makhov A.M., Conway J.F., Hafenstein S., Carson S.D. (2014). Kinetic and structural analysis of coxsackievirus B3 receptor interactions and formation of the A-particle. J. Virol..

[18] Xu L., Zheng Q., Zhu R., Yin Z., Yu H., Lin Y., Wu Y., He M., Huang Y., Jiang Y. (2021). Cryo-EM structures reveal the molecular basis of receptor-initiated coxsackievirus uncoating. Cell Host Microbe.

[19] Zhao X., Zhang G., Liu S., Chen X., Peng R., Dai L., Qu X., Li S., Song H., Gao Z. (2019). Human Neonatal Fc Receptor Is the Cellular Uncoating Receptor for Enterovirus B. Cell.

[20] Mattenberger F., Latorre V., Tirosh O., Stern A., Geller R. (2021). Globally defining the effects of mutations in a picornavirus capsid. eLife.

[21] Lefrancq N., Duret L., Bouchez V., Brisse S., Parkhill J., Salje H. (2025). Learning the fitness dynamics of pathogens from phylogenies. Nature.

[22] Haque S., Mathkor D.M., Alkhanani M.F., Bantun F., Momenah A.M., Faidah H., Jalal N.A., Kumar V. (2023). Comprehensive deep mutational scanning reveals the pH induced stability and binding differences between SARS-CoV-2 spike RBD and human ACE2. J. Biomol. Struct. Dyn..

[23] Alamri S.H., Haque S., Alghamdi B.S., Tayeb H.O., Azhari S., Farsi R.M., Elmokadem A., Alamri T.A., Harakeh S., Prakash A. (2025). Comprehensive mapping of mutations in TDP-43 and a-Synuclein that affect stability and binding. J. Biomol. Struct. Dyn..

[24] Dehury B., Raina V., Misra N., Suar M. (2021). Effect of mutation on structure, function and dynamics of receptor binding domain of human SARS-CoV-2 with host cell receptor ACE2: A molecular dynamics simulations study. J. Biomol. Struct. Dyn..

[25] Xue S., Han Y., Wu F., Wang Q. (2024). Mutations in the SARS-CoV-2 spike receptor binding domain and their delicate balance between ACE2 affinity and antibody evasion. Protein Cell.

[26] Pan J., Narayanan B., Shah S., Yoder J.D., Cifuente J.O., Hafenstein S., Bergelson J.M. (2011). Single amino acid changes in the virus capsid permit coxsackievirus B3 to bind decay-accelerating factor. J. Virol..

[27] Greaney A.J., Starr T.N., Gilchuk P., Zost S.J., Binshtein E., Loes A.N., Hilton S.K., Huddleston J., Eguia R., Crawford K.H.D. (2021). Complete Mapping of Mutations to the SARS-CoV-2 Spike Receptor-Binding Domain that Escape Antibody Recognition. Cell Host Microbe.

[28] Starr T.N., Greaney A.J., Hilton S.K., Ellis D., Crawford K.H.D., Dingens A.S., Navarro M.J., Bowen J.E., Tortorici M.A., Walls A.C. (2020). Deep Mutational Scanning of SARS-CoV-2 Receptor Binding Domain Reveals Constraints on Folding and ACE2 Binding. Cell.

[29] Hom N., Gentles L., Bloom J.D., Lee K.K. (2019). Deep Mutational Scan of the Highly Conserved Influenza A Virus M1 Matrix Protein Reveals Substantial Intrinsic Mutational Tolerance. J. Virol..

[30] Lei R., Hernandez Garcia A., Tan T.J.C., Teo Q.W., Wang Y., Zhang X., Luo S., Nair S.K., Peng J., Wu N.C. (2023). Mutational fitness landscape of human influenza H3N2 neuraminidase. Cell Rep..

[31] Haddox H.K., Dingens A.S., Hilton S.K., Overbaugh J., Bloom J.D. (2018). Mapping mutational effects along the evolutionary landscape of HIV envelope. eLife.

[32] Dingens A.S., Arenz D., Weight H., Overbaugh J., Bloom J.D. (2019). An Antigenic Atlas of HIV-1 Escape from Broadly Neutralizing Antibodies Distinguishes Functional and Structural Epitopes. Immunity.

[33] Álvarez-Rodríguez B., Buceta J., Geller R. (2023). Comprehensive profiling of neutralizing polyclonal sera targeting coxsackievirus B3. Nat. Commun..

[34] Álvarez-Rodríguez B., Velandia-Álvarez S., Toft C., Geller R. (2024). Mapping mutational fitness effects across the coxsackievirus B3 proteome reveals distinct profiles of mutation tolerability. PLoS Biol..

[35] Teng S., Sobitan A., Rhoades R., Liu D., Tang Q. (2021). Systemic effects of missense mutations on SARS-CoV-2 spike glycoprotein stability and receptor-binding affinity. Brief. Bioinform..

[36] Sharma A., Krishna S., Sowdhamini R. (2023). Bioinformatics Analysis of Mutations Sheds Light on the Evolution of Dengue NS1 Protein With Implications in the Identification of Potential Functional and Druggable Sites. Mol. Biol. Evol..

[37] Delgado J., Radusky L.G., Cianferoni D., Serrano L. (2019). FoldX 5.0: Working with RNA, small molecules and a new graphical interface. Bioinformatics.

[38] Sapozhnikov Y., Patel J.S., Ytreberg F.M., Miller C.R. (2023). Statistical modeling to quantify the uncertainty of FoldX-predicted protein folding and binding stability. BMC Bioinform..

[39] Usmanova D.R., Bogatyreva N.S., Ariño Bernad J., Eremina A.A., Gorshkova A.A., Kanevskiy G.M., Lonishin L.R., Meister A.V., Yakupova A.G., Kondrashov F.A. (2018). Self-consistency test reveals systematic bias in programs for prediction change of stability upon mutation. Bioinformatics.

[40] Delgado J., Reche R., Cianferoni D., Orlando G., van der Kant R., Rousseau F., Schymkowitz J., Serrano L. (2025). FoldX force field revisited, an improved version. Bioinformatics.

[41] Wang H., Fang Y., Jia Y., Tang J., Dong C. (2023). In silico epitope prediction and evolutionary analysis reveals capsid mutation patterns for enterovirus B. PLoS ONE.

[42] Rigsby R.E., Parker A.B. (2016). Using the PyMOL application to reinforce visual understanding of protein structure. Biochem. Mol. Biol. Educ..

[43] Smith D.J., Lapedes A.S., de Jong J.C., Bestebroer T.M., Rimmelzwaan G.F., Osterhaus A.D., Fouchier R.A. (2004). Mapping the antigenic and genetic evolution of influenza virus. Science.

[44] Han W., Zeng J., Shi J., Feng J., Chen Y., Zheng J., Cheng P., Zhai K., Zhang C., Qiu Z. (2025). Unraveling the mechanism behind the probable extinction of the B/Yamagata lineage of influenza B viruses. Nat. Commun..

[45] Li J., Wu Y.N., Zhang S., Kang X.P., Jiang T. (2022). Deep learning based on biologically interpretable genome representation predicts two types of human adaptation of SARS-CoV-2 variants. Brief. Bioinform..

[46] Sun Q., Shu C., Shi W., Luo Y., Fan G., Nie J., Bi Y., Wang Q., Qi J., Lu J. (2022). VarEPS: An evaluation and prewarning system of known and virtual variations of SARS-CoV-2 genomes. Nucleic Acids Res..

[47] Nicholls A., Sharp K.A., Honig B. (1991). Protein folding and association: Insights from the interfacial and thermodynamic properties of hydrocarbons. Proteins.

[48] Bueno M., Cremades N., Neira J.L., Sancho J. (2006). Filling small, empty protein cavities: Structural and energetic consequences. J. Mol. Biol..

[49] Starr T.N., Greaney A.J., Hannon W.W., Loes A.N., Hauser K., Dillen J.R., Ferri E., Farrell A.G., Dadonaite B., McCallum M. (2022). Shifting mutational constraints in the SARS-CoV-2 receptor-binding domain during viral evolution. Science.

[50] Starr T.N., Greaney A.J., Addetia A., Hannon W.W., Choudhary M.C., Dingens A.S., Li J.Z., Bloom J.D. (2021). Prospective mapping of viral mutations that escape antibodies used to treat COVID-19. Science.

[51] Cao Y., Wang J., Jian F., Xiao T., Song W., Yisimayi A., Huang W., Li Q., Wang P., An R. (2022). Omicron escapes the majority of existing SARS-CoV-2 neutralizing antibodies. Nature.

[52] Cao Y., Yisimayi A., Jian F., Song W., Xiao T., Wang L., Du S., Wang J., Li Q., Chen X. (2022). BA.2.12.1, BA.4 and BA.5 escape antibodies elicited by Omicron infection. Nature.

[53] Bloom J.D., Labthavikul S.T., Otey C.R., Arnold F.H. (2006). Protein stability promotes evolvability. Proc. Natl. Acad. Sci. USA.

[54] Álvarez-Rodríguez B., Bakhache W., McCormick L., Geller R., Dolan P.T. (2026). Comparative analysis of deep mutational scanning datasets in enteroviruses A and B identifies functional divergence and therapeutic targets. Nat. Ecol. Evol..

[55] Echave J., Wilke C.O. (2017). Biophysical models of protein evolution: Understanding the patterns of evolutionary sequence divergence. Annu. Rev. Biophys..

