# Supplementary material for: In Silico Saturation-Mutagenesis-Based Genomic Mutation Risk Assessment for Enterovirus B
Source: Viruses. 2026 Jun 3;18(6):645. doi: 10.3390/v18060645 (PMC13307921; doi:10.3390/v18060645)
Supplement: Supplementary file 1 [file viruses-18-00645-s001.zip › viruses-4301776-supplementary.pdf]

Table S1. Correlation of structural stability ΔΔG among EVB serotypes.

Serotype	CVB1	CVB3	E6	E30
CVB1	1	0.712*	0.731	0.739
CVB3	< 0.001#	1	0.680	0.751
E6	< 0.001	< 0.001	1	0.759
E30	< 0.001	< 0.001	< 0.001	1

* Spearman's correlation coefficients (upper triangle).

P values (lower triangle).

Table S2. Sites enhancing the structural stability of CVB1 after mutation.

ID	P1	Structural protein	Site	Amino acid	Secondary structure	Structural position	Average structural stability
1	64	VP4	64	T	Loop	Interface	-0.857
2	86	VP2	17	T	N-terminus	Interface	-0.554
3	119	VP2	50	E	N-terminus	Interface	-0.528
4	120	VP2	51	D	N-terminus	Interface	-0.641
5	164	VP2	95	N	Loop	Interface	-0.507
6	278	VP2	209	S	Loop	Interface	-0.860
7	288	VP2	219	N	Loop	Interface	-1.203
8	307	VP2	238	S	Loop	Interface	-0.566
9	338	VP3	6	T	N-terminus	Interface	-0.772
10	343	VP3	11	T	N-terminus	Interface	-1.107
11	382	VP3	50	D	N-terminus	Interface	-0.814
12	541	VP3	209	D	β sheet	Interface	-1.978
13	566	VP3	234	D	C-terminus	Outer surface	-1.521
14	610	VP1	40	S	N-terminus	Interface	-0.683
15	617	VP1	47	T	N-terminus	Interface	-1.068
16	627	VP1	57	H	N-terminus	Interface	-0.835
17	703	VP1	133	D	Loop	Interface	-2.111
18	707	VP1	137	Q	Loop	Core	-0.642
19	734	VP1	164	N	Loop	Interface	-0.596
20	740	VP1	170	T	β sheet	Interface	-0.537
21	767	VP1	197	T	Loop	Interface	-0.847
22	821	VP1	251	R	C-terminus	Interface	-0.585

Table S3. Sites enhancing the structural stability of CVB3 after mutation.

ID	P1	Structural protein	Site	Amino acid	Secondary structure	Structural position	Average structural stability
1	64	VP4	64	S	Loop	Interface	-0.841
2	119	VP2	50	E	N-terminus	Interface	-2.319
3	219	VP2	150	D	Loop	Interface	-0.684
4	233	VP2	164	S	Loop	Outer surface	-0.542
5	249	VP2	180	G	Loop	Interface	-0.608
6	307	VP2	238	T	Loop	Interface	-0.635
7	338	VP3	6	N	N-terminus	Interface	-0.971
8	478	VP3	146	V	Loop	Interface	-0.532
9	484	VP3	152	T	β sheet	Interface	-0.564
10	513	VP3	181	S	Loop	Outer surface	-0.640
11	529	VP3	197	N	Loop	Interface	-0.570
12	539	VP3	207	S	β sheet	Outer surface	-0.787
13	551	VP3	219	D	β sheet	Interface	-1.348
14	564	VP3	232	T	C-terminus	Interface	-0.720
15	589	VP1	19	G	N-terminus	Inner surface	-0.562
16	622	VP1	52	H	N-terminus	Interface	-0.554
17	647	VP1	77	T	β sheet	Outer surface	-0.906
18	669	VP1	99	Q	Loop	Interface	-0.783
19	768	VP1	198	E	Loop	Interface	-0.856
20	774	VP1	204	V	Loop	Outer surface	-1.037
21	804	VP1	234	R	β sheet	Interface	-1.054
22	840	VP1	270	T	C-terminus	Interface	-0.745

Table S4. Sites enhancing the structural stability of E6 after mutation.

ID	P1	Structural protein	Site	Amino acid	Secondary structure	Structural position	Average structural stability
1	7	VP4	7	T	Loop	Inner surface	-0.691
2	62	VP4	62	V	Loop	Inner surface	-0.582
3	84	VP2	15	S	N-terminus	Interface	-0.532
4	119	VP2	50	E	N-terminus	Interface	-1.429
5	120	VP2	51	D	N-terminus	Interface	-0.976
6	134	VP2	65	T	β sheet	Interface	-0.572
7	222	VP2	153	N	Loop	Outer surface	-0.614
8	287	VP2	218	N	Loop	Core	-0.906
9	363	VP3	33	E	N-terminus	Interface	-0.989
10	408	VP3	78	T	Loop	Interface	-0.762
11	423	VP3	93	T	Loop	Interface	-1.352
12	482	VP3	152	T	Loop	Interface	-0.809
13	549	VP3	219	D	C-terminus	Interface	-0.969
14	565	VP3	235	A	C-terminus	Outer surface	-1.188
15	598	VP1	30	A	N-terminus	Interface	-0.632
16	611	VP1	43	V	N-terminus	Interface	-0.522
17	613	VP1	45	S	N-terminus	Inner surface	-0.611
18	665	VP1	97	T	Loop	Outer surface	-0.721
19	684	VP1	116	D	β sheet	Interface	-0.921
20	704	VP1	136	D	Loop	Interface	-1.184
21	735	VP1	167	N	Loop	Interface	-0.628
22	741	VP1	173	T	β sheet	Interface	-0.752
23	796	VP1	228	T	Loop	Outer surface	-0.520
24	830	VP1	262	D	C-terminus	Outer surface	-0.591
25	842	VP1	274	S	C-terminus	Interface	-0.604

Table S5. Sites enhancing the structural stability of E30 after mutation.

ID	P1	Structural protein	Site	Amino acid	Secondary structure	Structural position	Average structural stability
1	64	VP4	64	T	Loop	Interface	-0.989
2	84	VP2	15	S	N-terminus	Inner surface	-0.596
3	109	VP2	40	T	N-terminus	Inner surface	-0.539
4	114	VP2	45	H	N-terminus	Interface	-0.547
5	153	VP2	84	E	Loop	Interface	-0.889
6	213	VP2	144	T	Loop	Outer surface	-0.847
7	325	VP2	256	R	C-terminus	Interface	-0.628
8	341	VP3	11	T	N-terminus	Interface	-0.834
9	380	VP3	50	D	N-terminus	Interface	-1.466
10	505	VP3	175	N	Loop	Interface	-1.002
11	510	VP3	180	T	Loop	Interface	-0.675
12	654	VP1	86	D	Loop	Outer surface	-0.501
13	666	VP1	98	T	Loop	Outer surface	-0.742
14	685	VP1	117	D	β sheet	Interface	-0.711
15	696	VP1	128	Q	β sheet	Interface	-0.870
16	787	VP1	219	H	β sheet	Outer surface	-0.580
17	832	VP1	264	N	C-terminus	Outer surface	-0.612
18	836	VP1	268	N	C-terminus	Interface	-0.565

Table S6. Correlation of receptor-binding ΔΔG among EVB serotypes.

Serotype	CVB1	CVB3	E6	E30
CVB1	1	0.317*	-0.057	-0.104
CVB3	<0.001#	1	0.031	-0.147
E6	0.107	0.387	1	0.051
E30	0.003	<0.001	0.151	1

* Spearman's correlation coefficients (upper triangle).

\# *P* values (lower triangle).

Table S7. Sites affecting CVB1 receptor-binding affinity after mutation.

ID	P1	Structural protein	Site	Amino acid	Secondary structure	Average receptor-binding affinity	Binding affinity grade	Risk score	Receptor-binding site
1	204	VP2	135	S	EF loop	-0.166	Enhanced	High	Proximal
2	205	VP2	136	N	EF loop	0.147	Decreased	Low	Yes
3	206	VP2	137	L	EF loop	-0.244	Enhanced	High	Proximal
4	207	VP2	138	N	EF loop	0.230	Decreased	Low	Yes
5	208	VP2	139	N	EF loop	1.333	Decreased	Low	Yes
6	209	VP2	140	T	EF loop	-0.443	Enhanced	High	Yes
7	211	VP2	142	E	EF loop	0.124	Decreased	Low	Proximal
8	214	VP2	145	E	EF loop	-0.225	Enhanced	High	Proximal
9	231	VP2	162	E	EF loop	-0.169	Enhanced	High	Proximal
10	234	VP2	165	A	EF loop	-0.435	Enhanced	High	Proximal
11	235	VP2	166	K	EF loop	0.802	Decreased	Low	Yes
12	512	VP3	180	V	GH loop	<0.001	Unchanged	Low	Yes
13	514	VP3	182	D	GH loop	<0.001	Unchanged	Low	Yes
14	515	VP3	183	E	GH loop	<0.001	Unchanged	Low	Yes
15	569	VP3	237	Y	C-terminus	-0.102	Enhanced	High	Spatially proximal
16	657	VP1	87	F	BC loop	-0.106	Enhanced	High	Proximal
17	659	VP1	89	E	C strand	-0.581	Enhanced	High	Yes
18	661	VP1	91	V	C strand	-0.094	Unchanged	Low	Yes
19	663	VP1	93	N	CD loop	-0.242	Enhanced	High	Proximal
20	715	VP1	145	P	EF loop	-0.454	Enhanced	High	Proximal
21	716	VP1	146	P	EF loop	-0.418	Enhanced	High	Yes
22	717	VP1	147	G	EF loop	-0.996	Enhanced	High	Yes
23	718	VP1	148	G	EF loop	-0.373	Enhanced	High	Yes
24	720	VP1	150	V	EF loop	0.719	Decreased	Low	Proximal
25	722	VP1	152	T	EF loop	-0.145	Enhanced	High	Proximal
26	772	VP1	202	N	GH loop	-0.116	Enhanced	High	Proximal
27	773	VP1	203	G	GH loop	-0.615	Enhanced	High	Yes
28	774	VP1	204	V	GH loop	-0.202	Enhanced	High	Yes
29	777	VP1	207	I	GH loop	-0.314	Enhanced	High	Proximal
30	779	VP1	209	T	GH loop	-0.452	Enhanced	High	Yes
31	782	VP1	212	N	GH loop	0.080	Unchanged	Low	Yes
32	785	VP1	215	T	H strand	1.305	Decreased	Low	Yes
33	787	VP1	217	Y	H strand	0.187	Decreased	Low	Yes
34	826	VP1	256	E	C-terminus	-0.227	Enhanced	High	Spatially proximal
35	827	VP1	257	K	C-terminus	0.311	Decreased	Low	Spatially proximal
36	828	VP1	258	Q	C-terminus	-0.459	Enhanced	High	Spatially proximal
37	829	VP1	259	K	C-terminus	-0.439	Enhanced	High	Spatially proximal
38	830	VP1	260	N	C-terminus	-0.471	Enhanced	High	Spatially proximal
39	832	VP1	262	N	C-terminus	-0.109	Enhanced	High	Spatially proximal
40	834	VP1	264	S	C-terminus	-0.114	Enhanced	High	Spatially proximal

Table S8. Sites affecting CVB3 receptor-binding affinity after mutation.

ID	P1	Structural protein	Site	Amino acid	Secondary structure	Average receptor-binding affinity	Binding affinity grade	Risk score	Receptor-binding site
1	203	VP2	134	C	EF loop	0.262	Decreased	Low	Proximal
2	205	VP2	136	T	EF loop	1.205	Decreased	Low	Yes
3	207	VP2	138	D	EF loop	-0.442	Enhanced	High	Yes
4	208	VP2	139	N	EF loop	1.699	Decreased	Low	Yes
5	209	VP2	140	T	EF loop	-0.017	Unchanged	Low	Yes
6	211	VP2	142	S	EF loop	0.108	Decreased	Low	Proximal
7	232	VP2	163	G	EF loop	-0.043	Unchanged	Low	Yes
8	233	VP2	164	S	EF loop	-0.165	Enhanced	High	Yes
9	234	VP2	165	N	EF loop	-0.011	Unchanged	Low	Yes
10	235	VP2	166	K	EF loop	0.602	Decreased	Low	Yes
11	657	VP1	87	Y	C strand	-0.187	Enhanced	High	Proximal
12	659	VP1	89	E	C strand	-0.979	Enhanced	High	Yes
13	660	VP1	90	W	C strand	0.001	Unchanged	Low	Yes
14	661	VP1	91	V	C strand	0.388	Decreased	Low	Yes
15	663	VP1	93	T	CD loop	-0.154	Enhanced	High	Proximal
16	665	VP1	95	R	CD loop	0.142	Decreased	Low	Proximal
17	713	VP1	143	Y	E strand	0.430	Decreased	Low	Proximal
18	715	VP1	145	P	EF loop	-0.272	Enhanced	High	Yes
19	716	VP1	146	P	EF loop	-0.544	Enhanced	High	Proximal
20	717	VP1	147	G	EF loop	-0.636	Enhanced	High	Proximal
21	718	VP1	148	G	EF loop	0.092	Unchanged	Low	Yes
22	720	VP1	150	V	EF loop	1.925	Decreased	Low	Proximal
23	722	VP1	152	D	EF loop	-0.293	Enhanced	High	Proximal
24	773	VP1	203	G	GH loop	-0.408	Enhanced	High	Proximal
25	774	VP1	204	V	GH loop	0.587	Decreased	Low	Proximal
26	778	VP1	208	N	GH loop	0.206	Decreased	Low	Proximal
27	781	VP1	211	N	GH loop	-0.174	Enhanced	High	Yes
28	782	VP1	212	N	GH loop	-0.624	Enhanced	High	Yes
29	783	VP1	213	M	GH loop	0.193	Decreased	Low	Yes
30	784	VP1	214	G	GH loop	0.785	Decreased	Low	Yes
31	785	VP1	215	T	H strand	0.449	Decreased	Low	Yes
32	787	VP1	217	Y	H strand	-0.188	Enhanced	High	Yes
33	829	VP1	259	K	C-terminus	0.501	Decreased	Low	Yes

Table S9. Sites affecting E6 receptor-binding affinity after mutation.

ID	P1	Structural protein	Site	Amino acid	Secondary structure	Average receptor-binding affinity	Binding affinity grade	Risk score	Receptor-binding site
1	203	VP2	134	A	EF loop	-0.179	Enhanced	High	Proximal
2	205	VP2	136	N	EF loop	0.125	Decreased	Low	Proximal
3	207	VP2	138	N	EF loop	-0.265	Enhanced	High	Yes
4	208	VP2	139	E	EF loop	0.128	Decreased	Low	Proximal
5	209	VP2	140	K	EF loop	0.160	Decreased	Low	Proximal
6	210	VP2	141	I	EF loop	-0.129	Enhanced	High	Proximal
7	233	VP2	164	N	EF loop	-0.540	Enhanced	High	Proximal
8	568	VP3	238	Q	C-terminus	0.184	Decreased	Low	Yes
9	643	VP1	75	Y	B strand	-0.591	Enhanced	High	Yes
10	645	VP1	77	V	B strand	-0.300	Enhanced	High	Proximal
11	654	VP1	86	P	BC loop	0.784	Decreased	Low	Proximal
12	655	VP1	87	D	BC loop	-0.641	Enhanced	High	Proximal
13	657	VP1	89	M	BC loop	-0.131	Enhanced	High	Proximal
14	658	VP1	90	Y	BC loop	0.340	Decreased	Low	Proximal
15	659	VP1	91	D	C strand	-0.524	Enhanced	High	Yes
16	660	VP1	92	S	C strand	-0.627	Enhanced	High	Yes
17	661	VP1	93	W	C strand	0.112	Decreased	Low	Proximal
18	662	VP1	94	V	C strand	-0.115	Enhanced	High	Proximal
19	667	VP1	99	Q	CD loop	1.122	Decreased	Low	Yes
20	672	VP1	104	R	CD loop	0.144	Decreased	Low	Proximal
21	718	VP1	150	G	EF loop	-0.030	Unchanged	Low	Yes
22	721	VP1	153	I	EF loop	0.803	Decreased	Low	Proximal
23	723	VP1	155	Q	EF loop	-0.366	Enhanced	High	Yes
24	724	VP1	156	A	EF loop	-0.419	Enhanced	High	Proximal
25	725	VP1	157	V	EF loop	-0.405	Enhanced	High	Proximal
26	727	VP1	159	D	EF loop	-0.824	Enhanced	High	Proximal
27	767	VP1	199	W	GH loop	0.494	Decreased	Low	Proximal
28	768	VP1	200	S	GH loop	0.526	Decreased	Low	Proximal
29	769	VP1	201	H	GH loop	0.801	Decreased	Low	Proximal
30	773	VP1	205	T	GH loop	0.251	Decreased	Low	Proximal
31	774	VP1	206	G	GH loop	5.291	Decreased	Low	Proximal
32	775	VP1	207	V	GH loop	0.209	Decreased	Low	Proximal
33	776	VP1	208	Y	GH loop	-0.113	Enhanced	High	Proximal
34	779	VP1	211	N	GH loop	-0.197	Enhanced	High	Yes
35	782	VP1	214	N	GH loop	-0.109	Enhanced	High	Proximal
36	790	VP1	222	R	H strand	-0.164	Enhanced	High	Proximal
37	827	VP1	259	T	C-terminus	-0.111	Enhanced	High	Proximal
38	828	VP1	260	H	C-terminus	-0.097	Unchanged	Low	Yes
39	829	VP1	261	K	C-terminus	1.640	Decreased	Low	Proximal
40	830	VP1	262	D	C-terminus	-0.465	Enhanced	High	Yes
41	833	VP1	265	D	C-terminus	0.172	Decreased	Low	Proximal

Table S10. Sites affecting E30 receptor-binding affinity after mutation.

ID	P1	Structural protein	Site	Amino acid	Secondary structure	Average receptor-binding affinity	Binding affinity grade	Risk score	Receptor-binding site
1	207	VP2	138	D	EF loop	-0.135	Enhanced	High	Yes
2	208	VP2	139	H	EF loop	-0.676	Enhanced	High	Yes
3	211	VP2	142	N	EF loop	-0.127	Enhanced	High	Yes
4	212	VP2	143	H	EF loop	-0.282	Enhanced	High	Proximal
5	213	VP2	144	T	EF loop	-0.135	Enhanced	High	Proximal
6	214	VP2	145	K	EF loop	-0.105	Enhanced	High	Proximal
7	231	VP2	162	D	EF loop	0.216	Decreased	Low	Spatially proximal
8	232	VP2	163	Q	EF loop	0.172	Decreased	Low	Spatially proximal
9	233	VP2	164	T	EF loop	0.192	Decreased	Low	Spatially proximal
10	234	VP2	165	G	EF loop	0.122	Decreased	Low	Spatially proximal
11	281	VP2	212	N	GH loop	0.105	Decreased	Low	Spatially proximal
12	284	VP2	215	R	GH loop	0.144	Decreased	Low	Spatially proximal
13	568	VP3	238	Q	C-terminus	0.068	Unchanged	Low	Yes
14	643	VP1	75	Y	B strand	-0.185	Enhanced	High	Proximal
15	645	VP1	77	A	B strand	-0.145	Enhanced	High	Proximal
16	648	VP1	80	A	B strand	-0.140	Enhanced	High	Proximal
17	651	VP1	83	K	BC loop	-0.131	Enhanced	High	Proximal
18	653	VP1	85	N	BC loop	-0.733	Enhanced	High	Proximal
19	654	VP1	86	D	BC loop	-1.612	Enhanced	High	Yes
20	655	VP1	87	E	BC loop	-0.250	Enhanced	High	Yes
21	656	VP1	88	L	BC loop	-0.348	Enhanced	High	Proximal
22	657	VP1	89	D	BC loop	-0.410	Enhanced	High	Proximal
23	660	VP1	92	T	C strand	-0.030	Unchanged	Low	Yes
24	661	VP1	93	N	C strand	0.453	Decreased	Low	Yes
25	719	VP1	151	G	EF loop	-0.055	Unchanged	Low	Yes
26	722	VP1	154	I	EF loop	1.040	Decreased	Low	Proximal
27	724	VP1	156	K	EF loop	0.082	Unchanged	Low	Yes
28	725	VP1	157	S	EF loop	-0.604	Enhanced	High	Proximal
29	728	VP1	160	D	EF loop	0.216	Decreased	Low	Proximal
30	731	VP1	163	W	EF loop	0.149	Decreased	Low	Proximal
31	767	VP1	199	G	GH loop	0.167	Decreased	Low	Proximal
32	769	VP1	201	S	GH loop	0.298	Decreased	Low	Proximal
33	770	VP1	202	H	GH loop	-0.049	Unchanged	Low	Yes
34	774	VP1	206	S	GH loop	-0.266	Enhanced	High	Proximal
35	775	VP1	207	G	GH loop	3.978	Decreased	Low	Yes
36	776	VP1	208	V	GH loop	1.130	Decreased	Low	Proximal
37	779	VP1	211	Y	GH loop	0.125	Decreased	Low	Proximal
38	780	VP1	212	T	GH loop	0.475	Decreased	Low	Yes
39	781	VP1	213	T	GH loop	0.403	Decreased	Low	Proximal
40	782	VP1	214	L	GH loop	0.283	Decreased	Low	Proximal
41	783	VP1	215	N	GH loop	0.185	Decreased	Low	Proximal
42	784	VP1	216	N	GH loop	0.340	Decreased	Low	Proximal
43	789	VP1	221	Y	H strand	0.191	Decreased	Low	Proximal
44	829	VP1	261	K	C-terminus	-0.209	Enhanced	High	Proximal
45	830	VP1	262	A	C-terminus	-0.132	Enhanced	High	Proximal
46	831	VP1	263	R	C-terminus	-0.499	Enhanced	High	Yes
47	832	VP1	264	N	C-terminus	-0.328	Enhanced	High	Proximal

Table S11. High-risk sites for fitness of CVB3.

ID	P1	Structural protein	Site	Amino acid	ID	P1	Structural protein	Site	Amino acid
1	25	VP4	25	I	42	567	VP3	235	N
2	47	VP4	47	T	43	579	VP1	9	A
3	112	VP2	43	K	44	580	VP1	10	A
4	114	VP2	45	S	45	581	VP1	11	I
5	139	VP2	70	Q	46	583	VP1	13	R
6	143	VP2	74	T	47	585	VP1	15	A
7	145	VP2	76	P	48	588	VP1	18	V
8	206	VP2	137	L	49	589	VP1	19	G
9	207	VP2	138	D	50	590	VP1	20	T
10	211	VP2	142	S	51	593	VP1	23	T
11	212	VP2	143	S	52	596	VP1	26	E
12	213	VP2	144	A	53	597	VP1	27	A
13	220	VP2	151	S	54	608	VP1	38	H
14	222	VP2	153	K	55	615	VP1	45	G
15	225	VP2	156	A	56	622	VP1	52	H
16	227	VP2	158	K	57	624	VP1	54	K
17	230	VP2	161	A	58	650	VP1	80	K
18	231	VP2	162	S	59	652	VP1	82	S
19	233	VP2	164	S	60	654	VP1	84	A
20	235	VP2	166	K	61	655	VP1	85	K
21	236	VP2	167	L	62	661	VP1	91	V
22	330	VP2	261	G	63	696	VP1	126	P
23	331	VP2	262	H	64	698	VP1	128	T
24	367	VP3	35	R	65	699	VP1	129	T
25	373	VP3	41	K	66	700	VP1	130	Q
26	391	VP3	59	G	67	720	VP1	150	V
27	395	VP3	63	N	68	723	VP1	153	K
28	401	VP3	69	Q	69	767	VP1	197	S
29	408	VP3	76	E	70	770	VP1	200	S
30	410	VP3	78	S	71	772	VP1	202	N
31	423	VP3	91	Y	72	774	VP1	204	V
32	473	VP3	141	A	73	777	VP1	207	I
33	475	VP3	143	T	74	800	VP1	230	K
34	476	VP3	144	K	75	824	VP1	254	Q
35	512	VP3	180	A	76	827	VP1	257	K
36	513	VP3	181	S	77	829	VP1	259	K
37	537	VP3	205	Q	78	834	VP1	264	Q
38	538	VP3	206	S	79	837	VP1	267	G
39	541	VP3	209	Y	80	840	VP1	270	T
40	564	VP3	232	S	81	841	VP1	271	T
41	566	VP3	234	Q	82	843	VP1	273	Q

Table S12. Bioinformatics characteristics of CVB3 [n (%)].

Characteristic	High-risk sites	Low-risk sites	Total*
Structural protein			
VP4	2 (2.4)	53 (7.2)	55 (6.7)
VP2	21 (25.6)	235 (31.8)	256 (31.2)
VP3	19 (23.2)	219 (29.6)	238 (29.0)
VP1	40 (48.8)	232 (31.4)	272 (33.1)
Information entropy (genotype)			
Low	2 (2.4)	403 (54.5)	405 (49.3)
Medium	9 (11.0)	202 (27.4)	211 (25.7)
High	71 (86.6)	134 (18.1)	205 (25.0)
Information entropy (serotype)			
Low	52 (63.4)	684 (92.6)	736 (89.6)
High	30 (36.6)	55 (7.4)	85 (10.4)
Secondary structure			
Loop	42 (51.2)	321 (43.5)	363 (44.2)
β Sheet	5 (6.1)	213 (28.8)	218 (26.5)
N-terminus	22 (26.8)	165 (22.3)	187 (22.8)
C-terminus	13 (15.9)	40 (5.4)	53 (6.5)
Structural position			
Core	1 (1.2)	142 (19.2)	143 (17.4)
Interface	41 (50.0)	520 (70.4)	561 (68.3)
Outer surface	34 (41.5)	61 (8.2)	95 (11.6)
Inner surface	6 (7.3)	16 (2.2)	22 (2.7)
Distance from the α-carbon to the center			
Close to the center	21 (25.6)	183 (24.8)	204 (24.8)
Medium distance	10 (12.2)	402 (54.4)	412 (50.2)
Far from the center	51 (62.2)	154 (20.8)	205 (25.0)
Relative surface accessibility			
Low	1 (1.2)	408 (55.2)	409 (49.8)
Medium	20 (24.4)	187 (25.3)	207 (25.2)
High	61 (74.4)	144 (19.5)	205 (25.0)
Antigenic epitope			
Yes	46 (56.1)	143 (19.4)	189 (23.0)
No	36 (43.9)	596 (80.6)	632 (77.0)
Receptor-binding site			
Yes	5 (6.1)	15 (2.0)	20 (2.4)
No	77 (93.9)	724 (98.0)	801 (97.6)
Total	82	739	821

* Thirty sites of fitness were missing.

Table S13. Prediction results of the EVB fitness risk prediction model.

Datasets	Categories	Site numbers	Recall (%)	Precision (%)	F1-score (%)
Training datasets	High-risk sites	520	100.0	94.7	97.3
Training datasets	Low-risk sites	514	94.4	100.0	97.1
Test datasets	High-risk sites	219	100.0	93.2	96.5
Test datasets	Low-risk sites	225	92.9	100.0	96.3

Table S14. Correlation of fitness risk scores among EVB serotypes.

Serotype	CVB1	CVB3	E6	E30
CVB1	1	0.533*	0.374	0.668
CVB3	< 0.001#	1	0.242	0.590
E6	< 0.001	< 0.001	1	0.322
E30	< 0.001	< 0.001	< 0.001	1

* Phi (φ) coefficient (upper triangle).

P values (lower triangle).

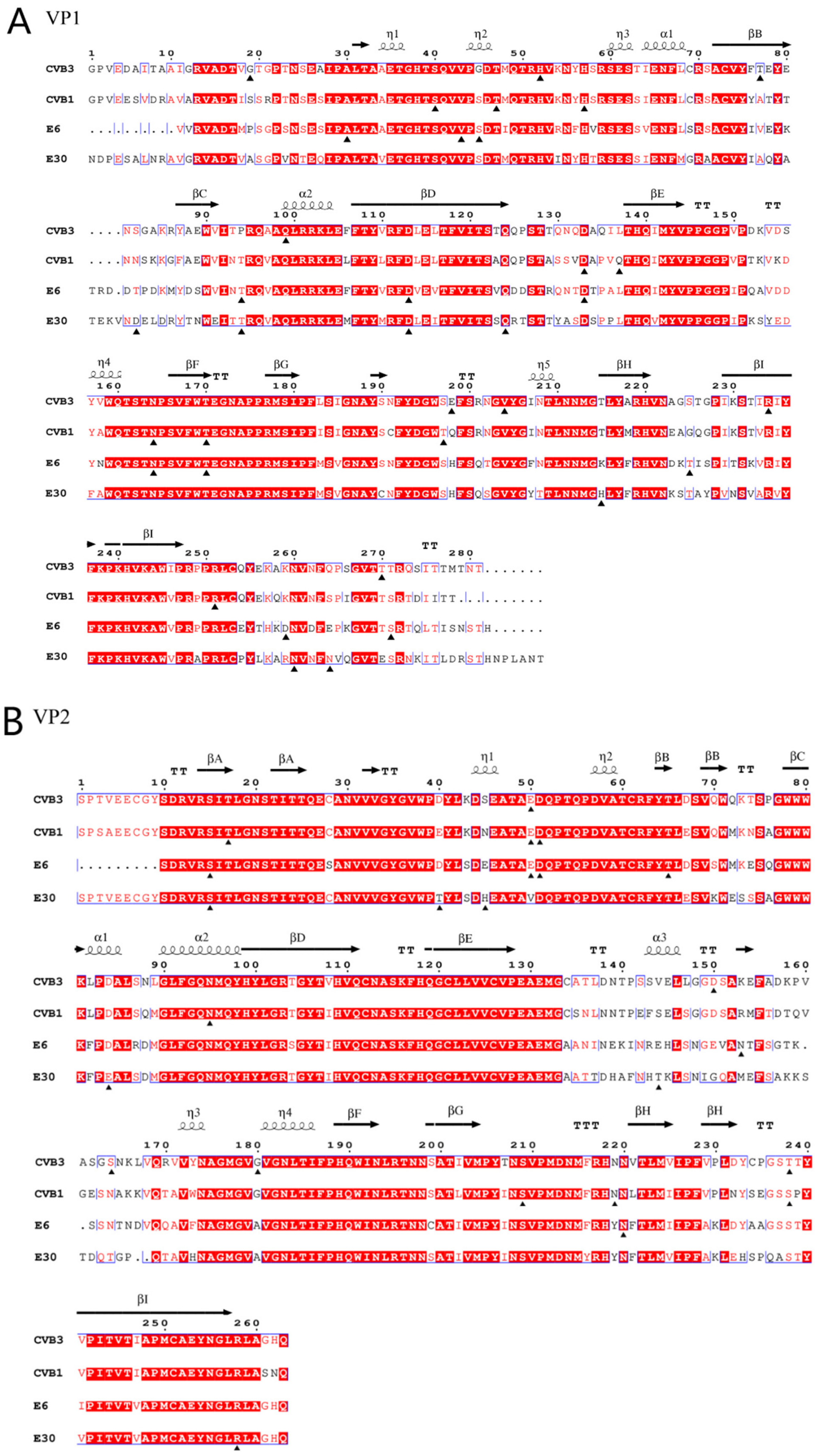
A
VP1
CVB3
CVB1
E6
E30
B
VP2
CVB3
CVB1
E6
E30

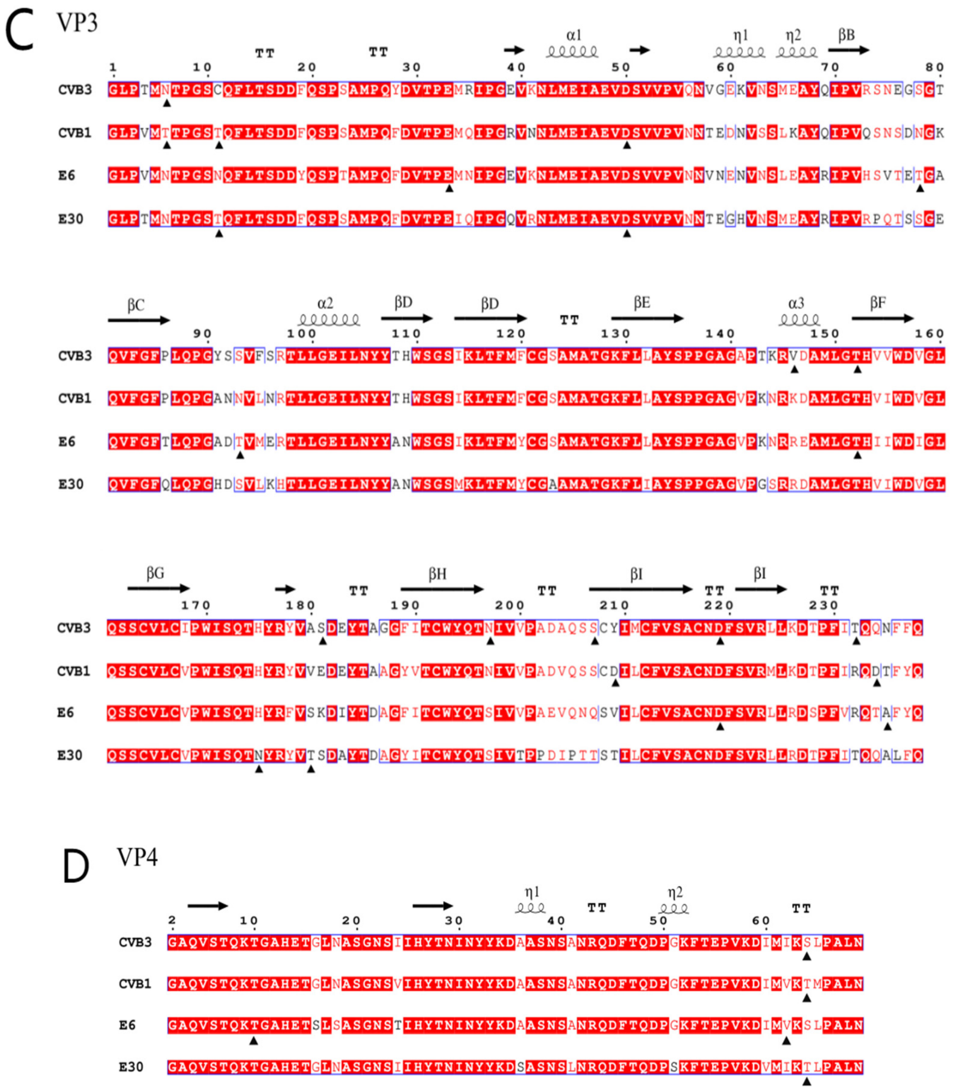

Figure S1. Sequence alignment of high-risk sites for structural stability of EVB. The secondary structural elements of VP1 (**A**), VP2 (**B**), VP3 (**C**), and VP4 (**D**) are labeled. Triangles indicate high-risk sites.

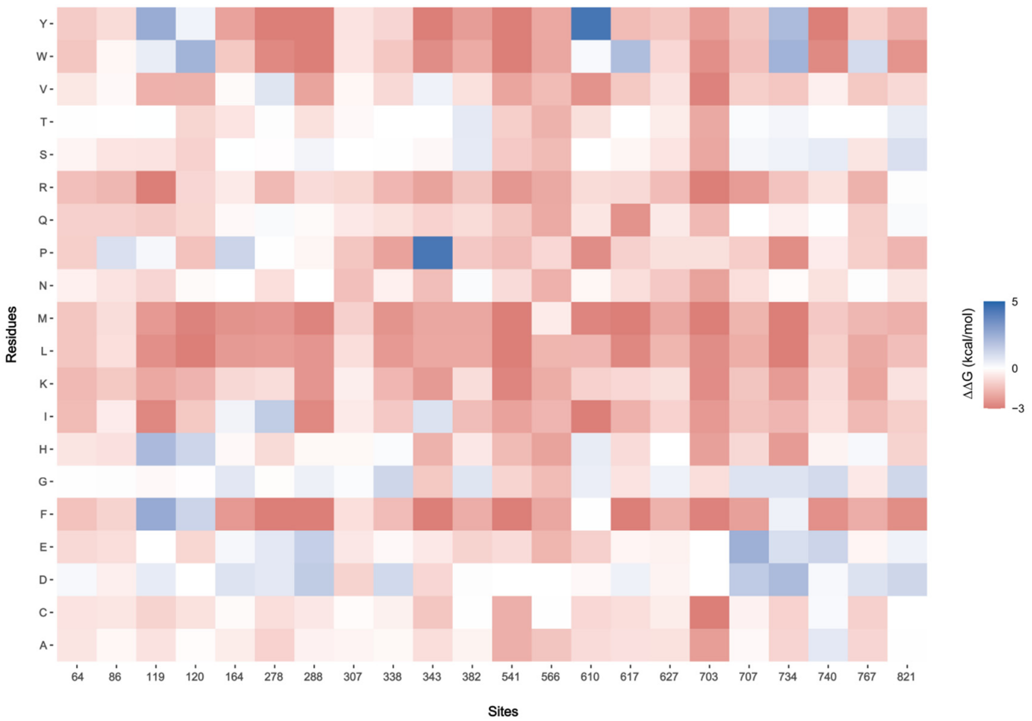

Figure S2. Heatmap of sites enhancing the structural stability of CVB1 after mutation.

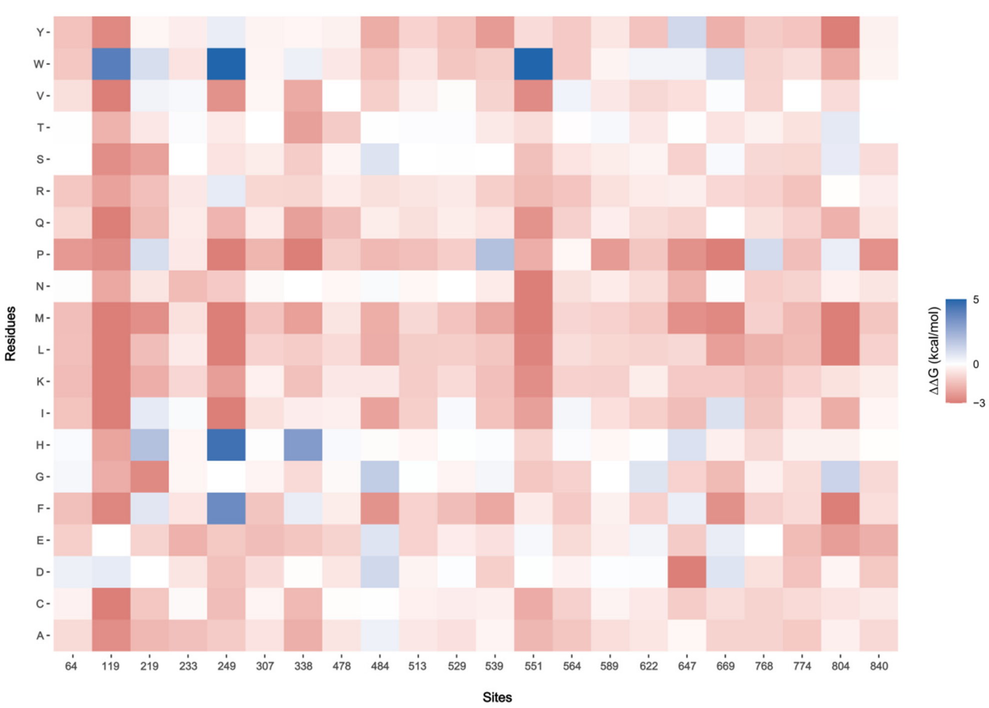

Figure S3. Heatmap of sites enhancing the structural stability of E6 after mutation.

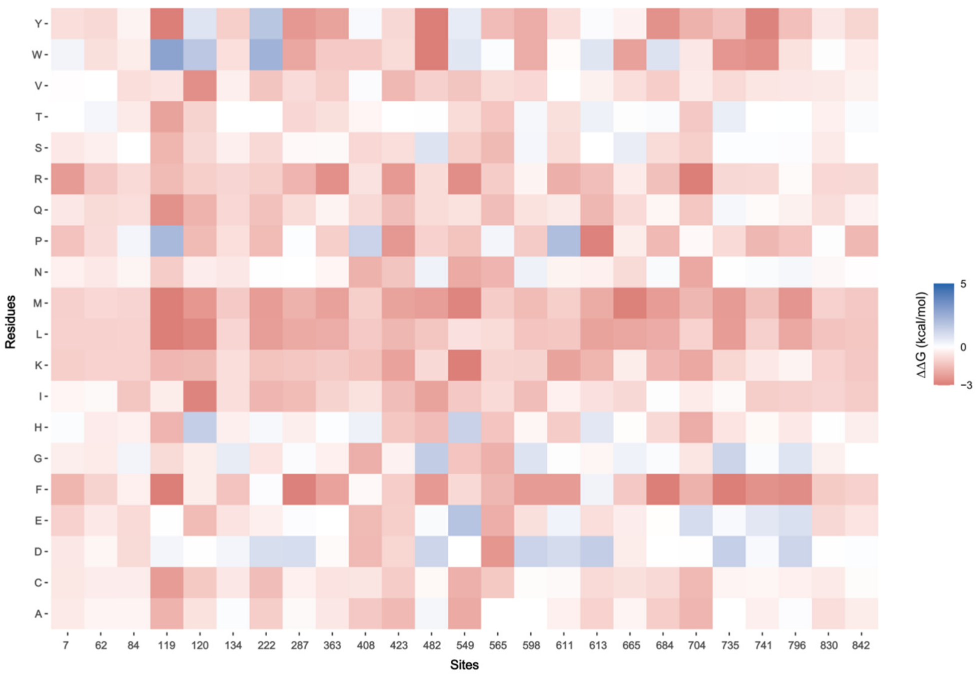

Figure S4. Heatmap of sites enhancing the structural stability of E6 after mutation.

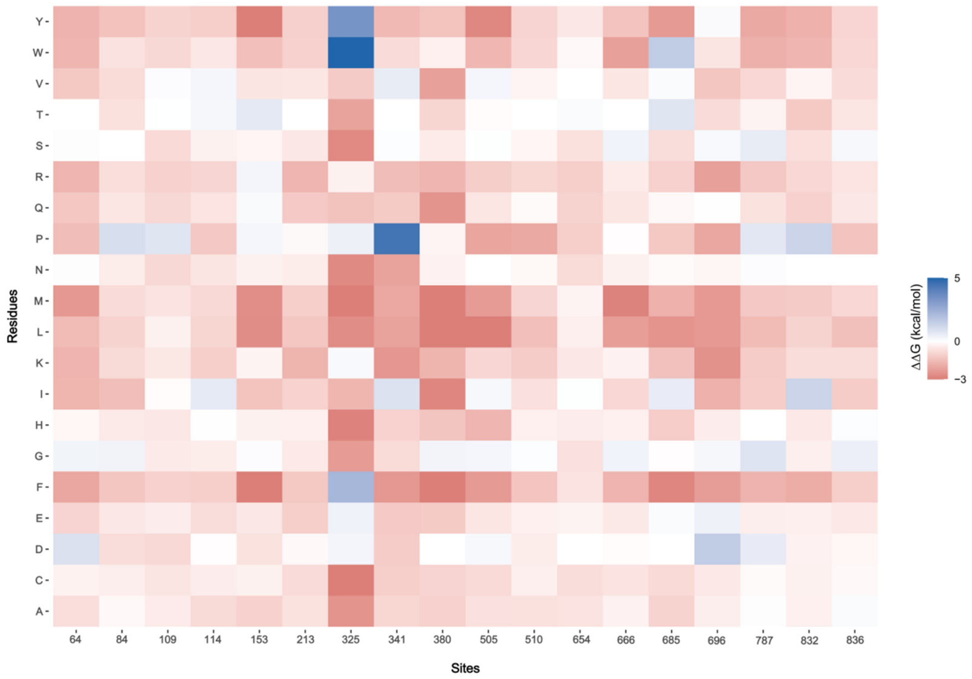

Figure S5. Heatmap of sites enhancing the structural stability of E30 after mutation.

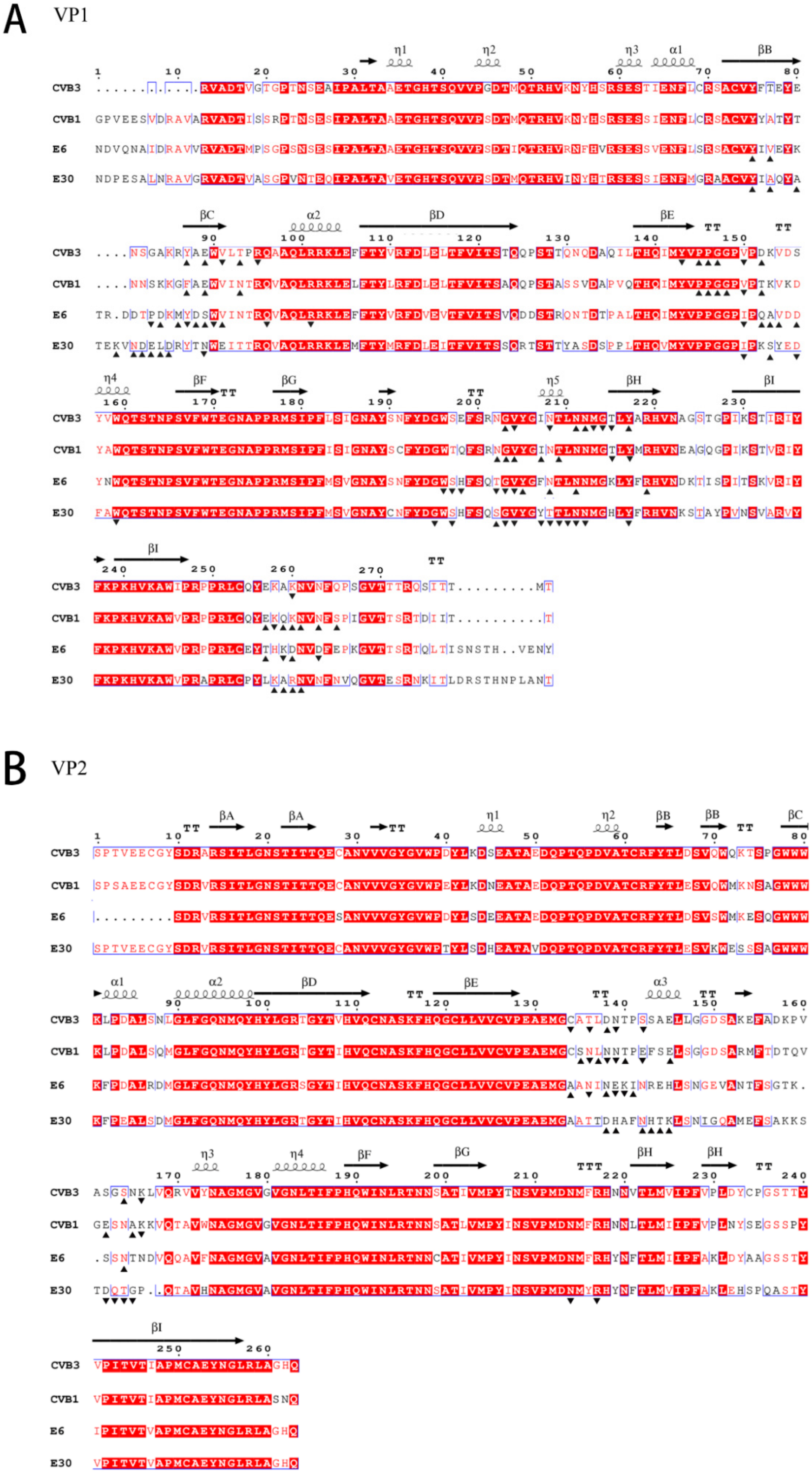
A
VP1
CVB3
CVB1
E6
E30
B
VP2
CVB3
CVB1
E6
E30

C VP3

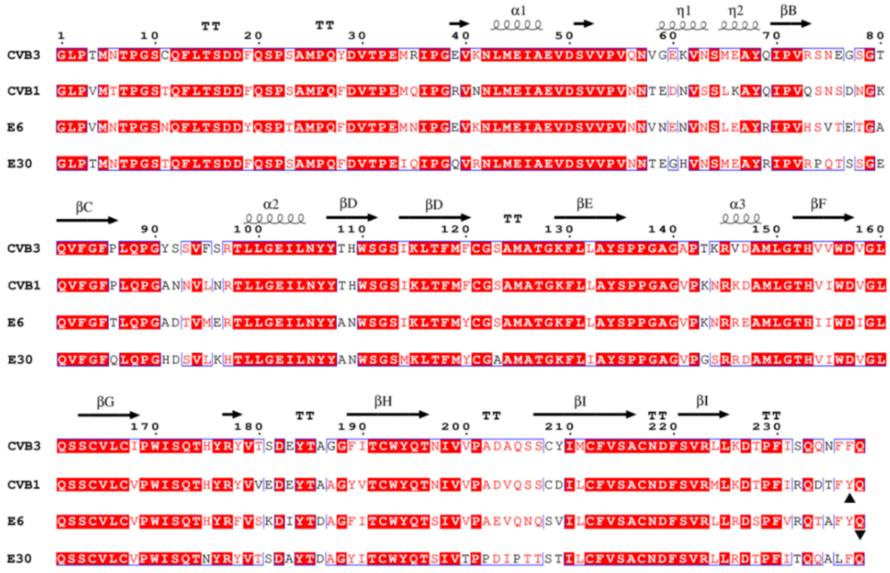

Figure S6. Sequence alignment of high-risk sites for receptor-binding affinity of EVB. The secondary structural elements of VP1 (**A**), VP2 (**B**), and VP3 (**C**) are labeled. Upward and downward triangles indicate sites that enhance and reduce receptor-binding affinity, respectively.

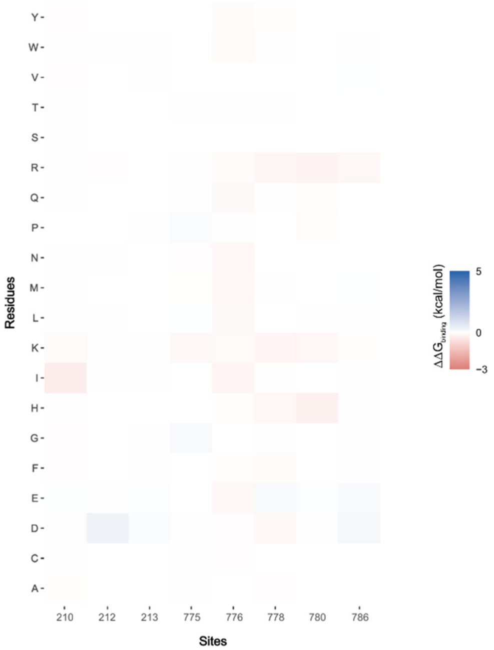

Figure S7. Computational saturation mutagenesis on receptor-binding affinity of partial sites in CVB1.

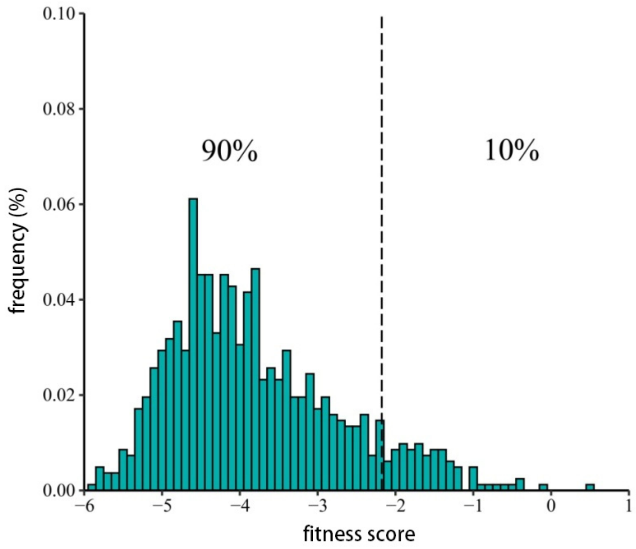

Figure S8. Frequency distribution of CVB3 fitness scores. The fitness scores of CVB3 show a skewed distribution. The 90th percentile of the fitness scores was used to classify risk. Sites with scores higher than the percentile were defined as high-risk sites, and those with scores lower than the percentile were defined as low-risk sites.

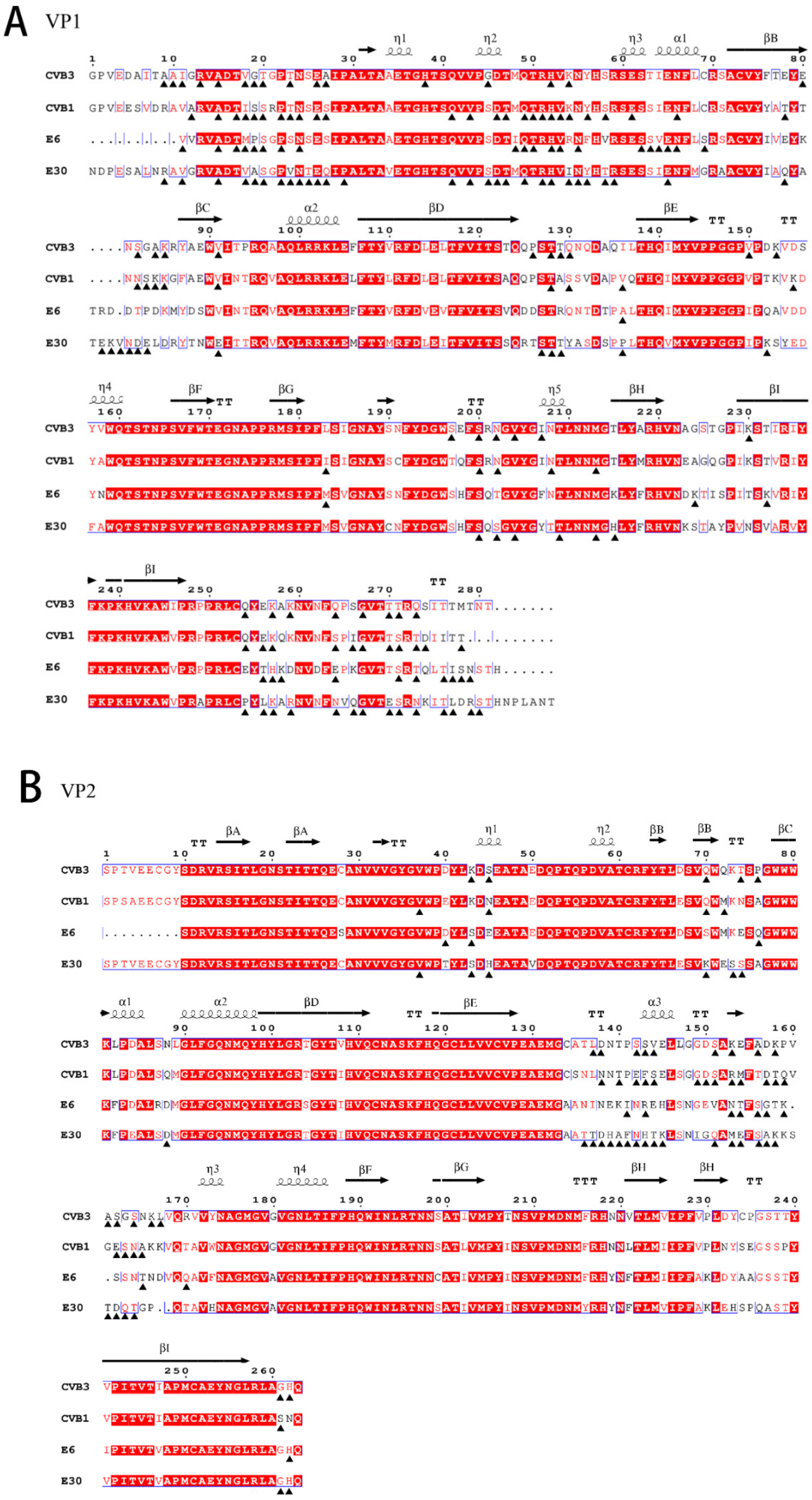
A
VP1
CVB3
CVB1
E6
E30
B
VP2
CVB3
CVB1
E6
E30

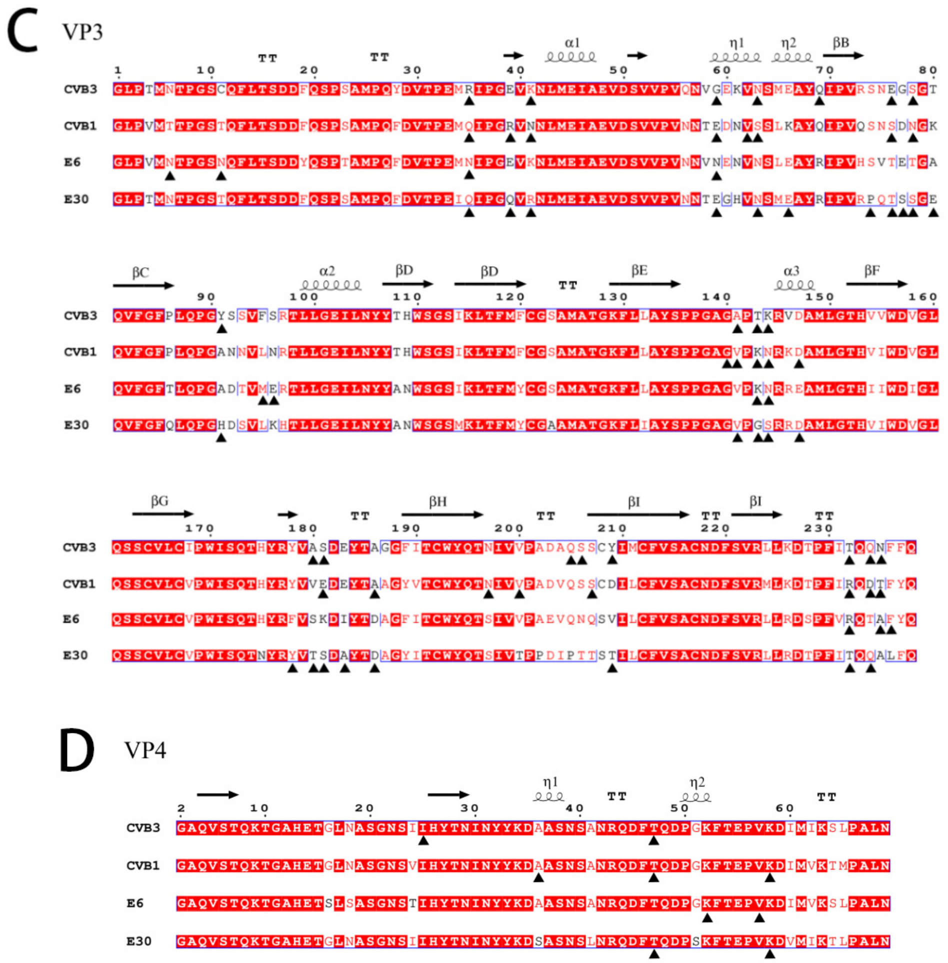

Figure S9. Sequence alignment of high-risk sites for fitness of EVB. The secondary structural elements of VP1 (**A**), VP2 (**B**), VP3 (**C**), and VP4 (**D**) are labeled. Triangles indicate high-risk sites.

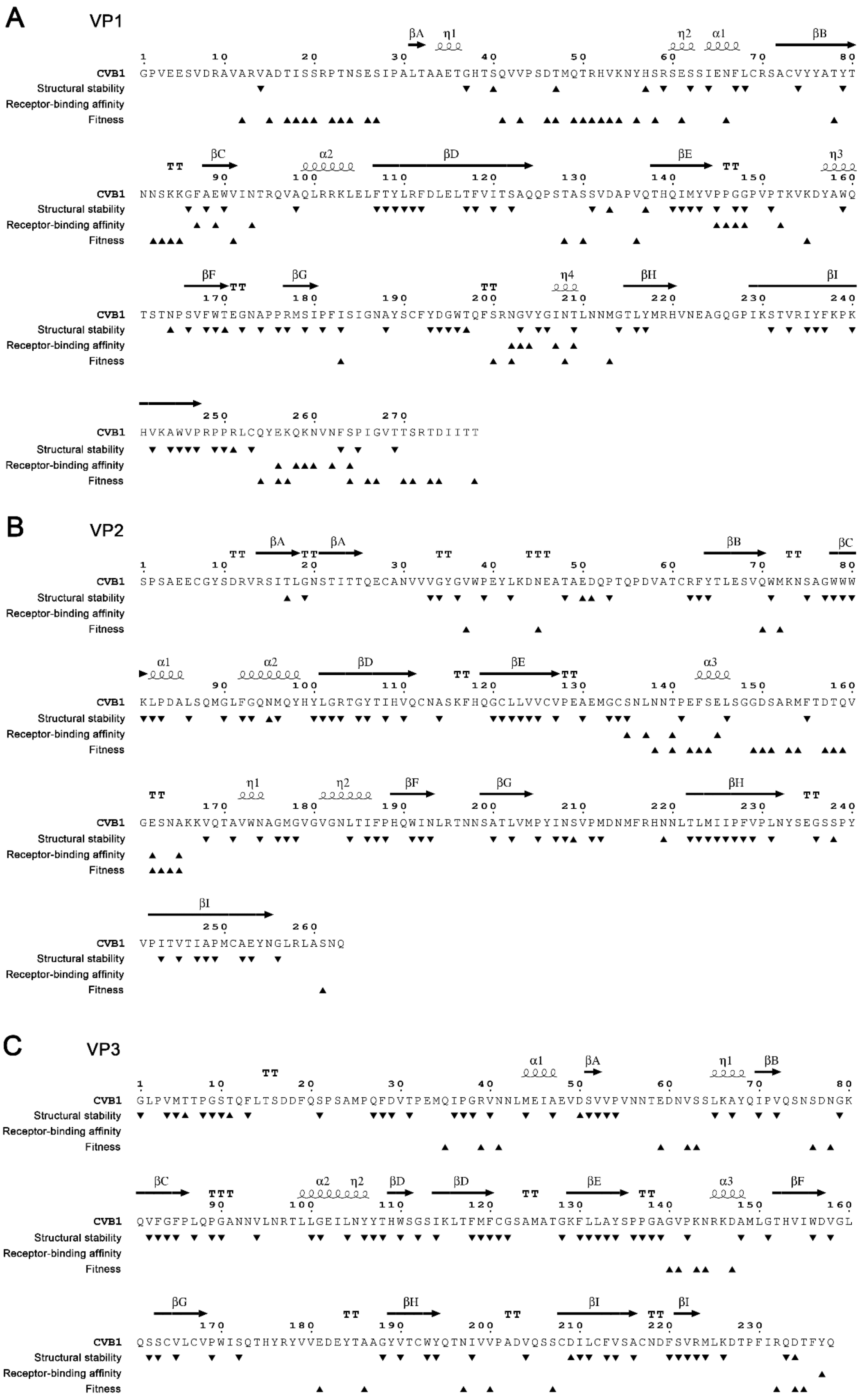

A
VP1
CVB1
GPVEESVDRAVARVADTISSRPTNSESIPALTAAETGHTSQVVPSDTMQTRHVKNYHSRSESSIENFLCRSACVYYATYT
NNSKKGFAEWVINTRQVAQLRRKLELFTYLRFDLELTFVITSAQQPSTASSVDAPVQTHQIMYVPPGGPVPTKVKDYAWQ
TSTNPSVFWTEGNAPPRMSIPFISIGNAYSCFYDGWTQFSRNGVYGINTLNNMGTLYMRHVNEAGQGPIKSTVRIYFKPK
HVKAWVPRPPRLCQYEKQKNVNFSPIGVTTSRTDIITT
Structural stability
Receptor-binding affinity
Fitness
B
VP2
CVB1
SPSAEECGYSDRVRSITLGNSTITTQECANVVVGYGVWPEYLKDNEATAEDQPTQPDVATCRFYTLESVQWMKNSAGWWW
KLPDALSQMGLFGQNMQYHYLGRTGYTIHVQCNASKFHQGCLLVVCVPEAEMGCSNLNNTPEFSELSGGDSARMFTDTQV
GESNAKKVQTAVWNAGMGVGVGNLTIFPHQWINLRTNNSATLVMPYINSVPMDNMFRHNNLTLMIIPFVPLNYSEGSSPY
VPITVTIAPMCAEYNGLRLASNQ
Structural stability
Receptor-binding affinity
Fitness
C
VP3
CVB1
GLPVMTTPGSTQFLTSDDFQSPSAMPQFDVTPEMQIPGRVNNLMEIAEVDSVVPVNNTEDNVSSLKAYQIPVQSNSDNGK
QVFGFPLQPGANNVLNRTLLGEILNYYTHWSGSIKLTFMFCGSAMATGKFLLAYSPPGAGVPKNRKDAMLGTHVIWDVGL
QSSCVLCVPWISQTHYRYVVEDEYTAAGYVTCWYQTNIVVPADVQSSCDILCFVSACNDFSVRMLKDTPFIRQDTFYQ
Structural stability
Receptor-binding affinity
Fitness

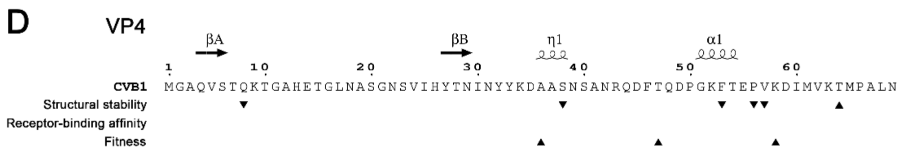

Figure S10. Sequence alignment of risk sites for CVB1. The first row shows the computational saturation mutagenesis results for structural stability. The upper triangle indicates mutations that enhance structural stability (high-risk sites). The lower triangle indicates mutations that greatly reduce structural stability (extremely low-risk sites). The second row shows the computational saturation mutagenesis results for receptor-binding affinity. The upper triangle indicates mutations that enhance receptor-binding affinity (high-risk sites). The third row shows the computational saturation mutagenesis results for fitness. The upper triangle indicates mutations that enhance fitness (high-risk sites).

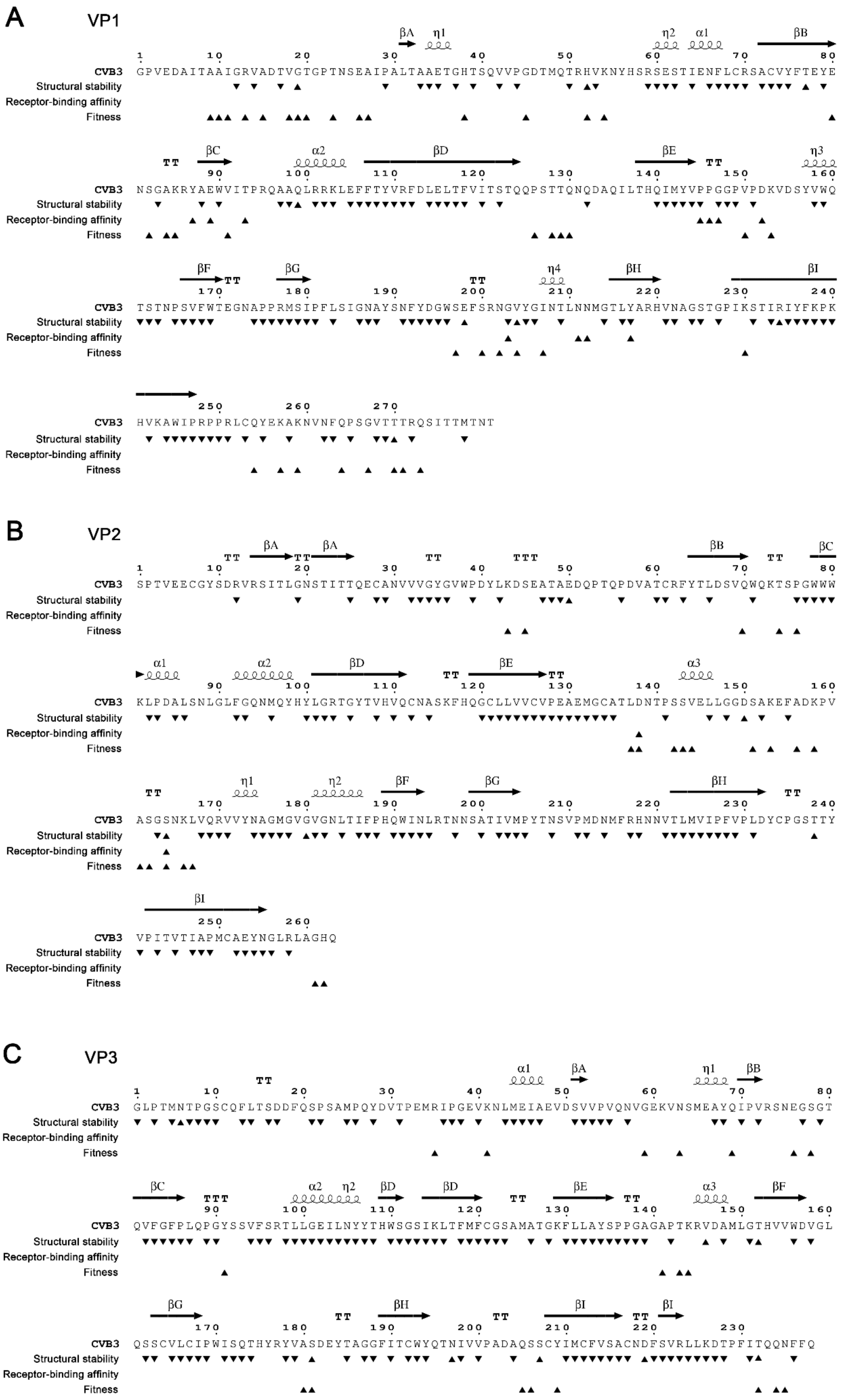
A
VP1
CVB3 GPVEDAITAAIGRVADTVGTGPTNSEAIPALTAAETGHTSQVVPGDTMQTRHVKNYHSRSESTIENFLCRSACVYFTEYE
CVB3 NSGAKRYAEWVITPRQAAQLRRKLEFFTYVRFDLELTFVITSTQQPSTTQNQDAQILTHQIMYVPPGGPVPDKVDSYVWQ
CVB3 TSTNPSVFWTEGNAPPRMSIPFLSIGNAYSNFYDGWSEFSRNGVYGINTLNNMGTLYARHVNAGSTGPIKSTIRIYFKPK
CVB3 HVKAWIPRPPRLCQYEKAKNVNFQPSGVTTTRQSITTMTNT
Structural stability
Receptor-binding affinity
Fitness
B
VP2
CVB3 SPTVEECGYSDRVRSITLGNSTITTQECANVVVGYGVWPDYLKDSEATAEDQPTQPDVATCRFYTLDSVQWQKTSPGWWW
CVB3 KLPDALSNLGLFGQNMQYHYLGRTGYTVHVQCNASKFHQGCLLVVCVPEAEMGCATLDNTPSSVELLGGDSAKEFADKPV
CVB3 ASGSNKLVQRVVYNAGMGVGVGNLTIFPHQWINLRTNNSATIVMPYTNSVPMDNMFRHNNVTLMVIPFVPLDYCPGSTTY
CVB3 VPITVTIAPMCAEYNGLRLAGHQ
C
VP3
CVB3 GLPTMNTPGSCQFLTSDDFQSPSAMPQYDVTPEMRIPGEVKNLMEIAEVDSVVPVQNVGEKVNSMEAYQIPVRSNEGSGT
CVB3 QVFGFPLQPGYSSVFSRTLLGEILNYYTHWSGSIKLTFMFCGSAMATGKFLLAYSPPGAGAPTKRVDAMLGTHVVWDVGL
CVB3 QSSCVLCIPWISQTHYRYVASDEYTAGGFITCWYQTNIVVPADAQSSCYIMCFVSACNDFSVRLLKDTPFITQQNFFQ

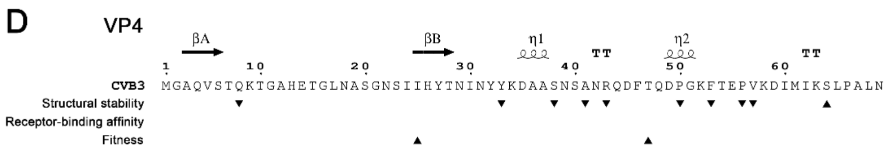

Figure S11. Sequence alignment of risk sites for CVB3.

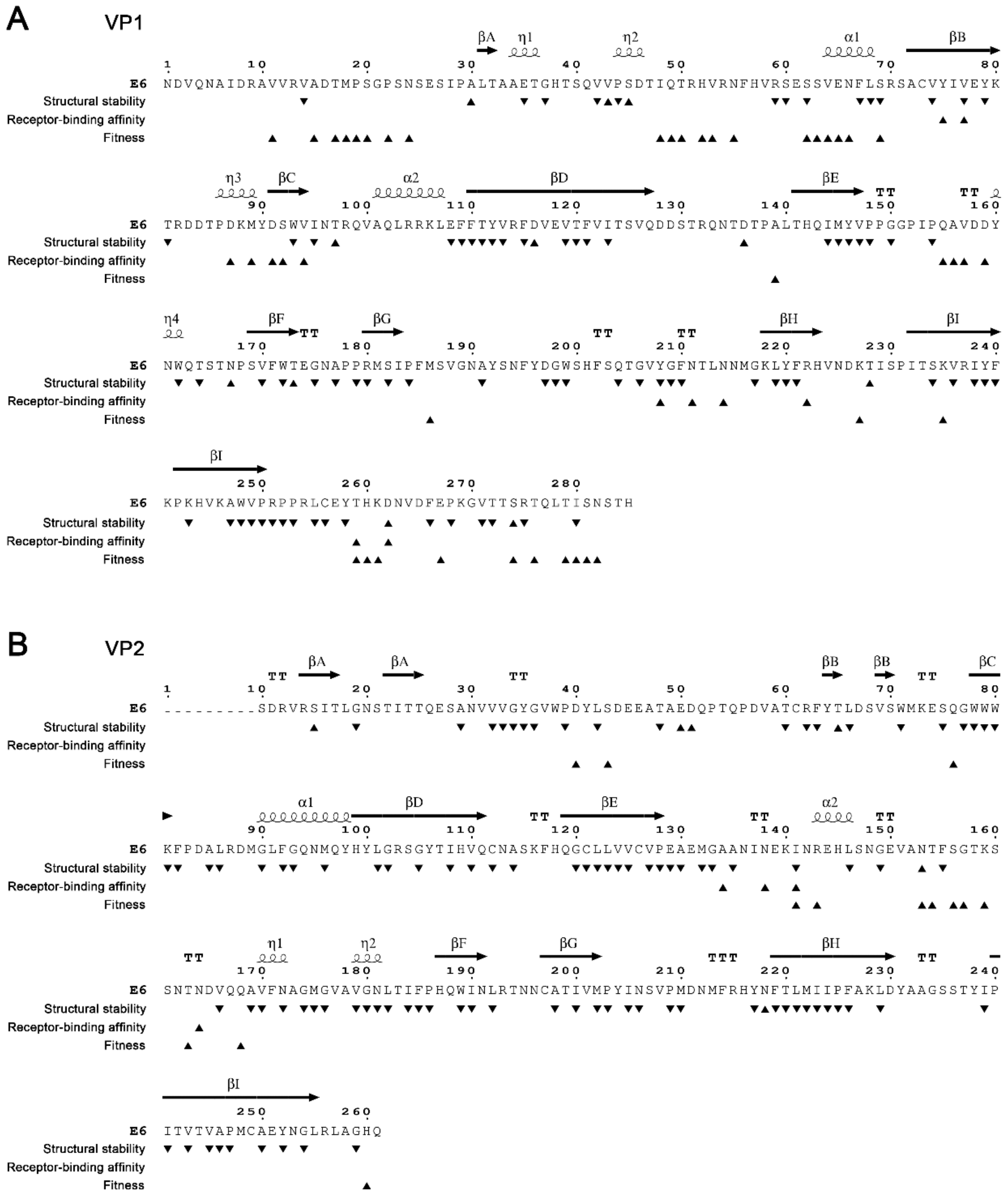

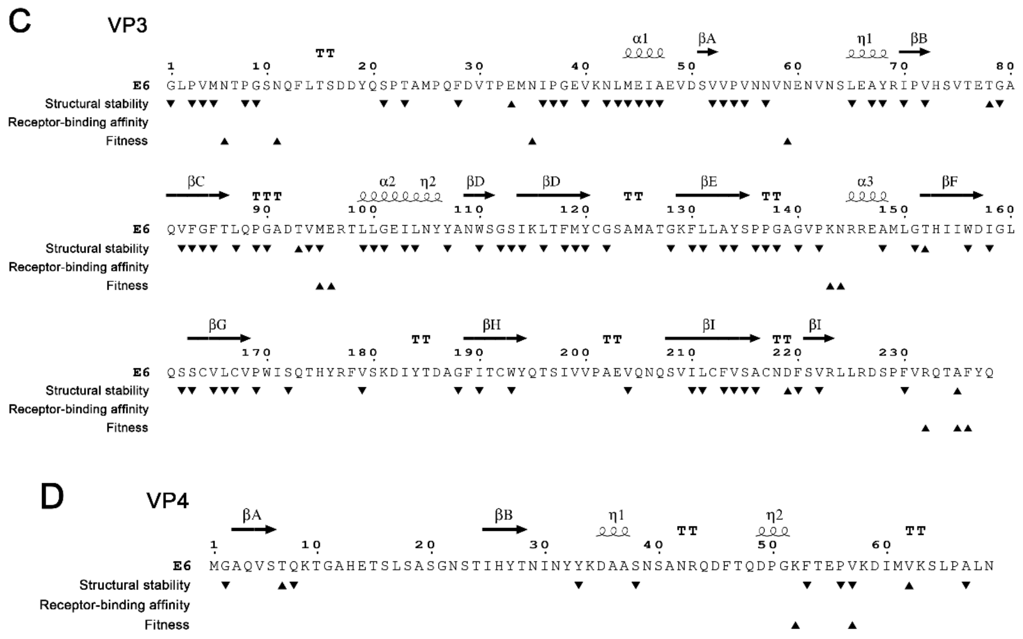

Figure S12. Sequence alignment of risk sites for E6.

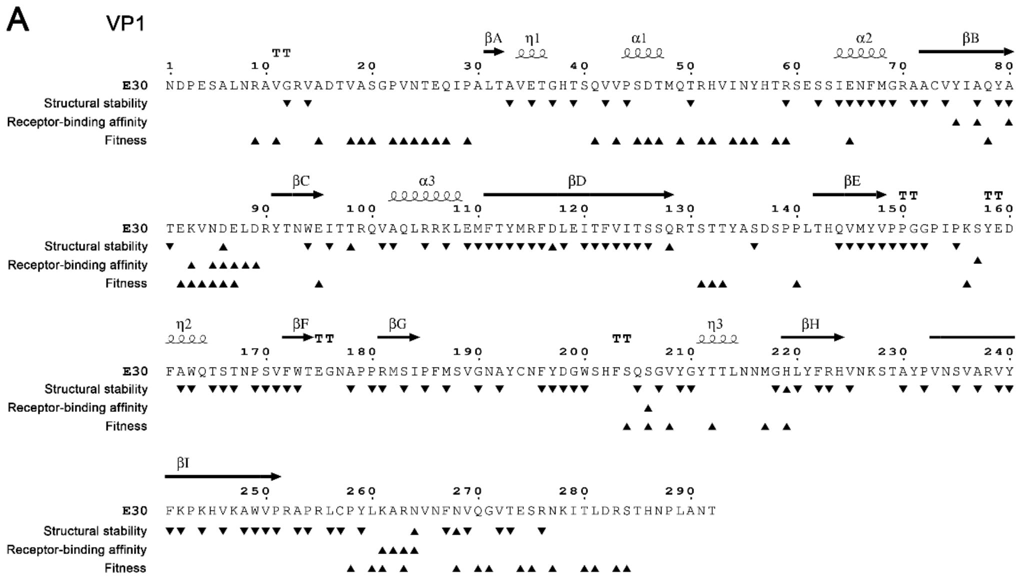

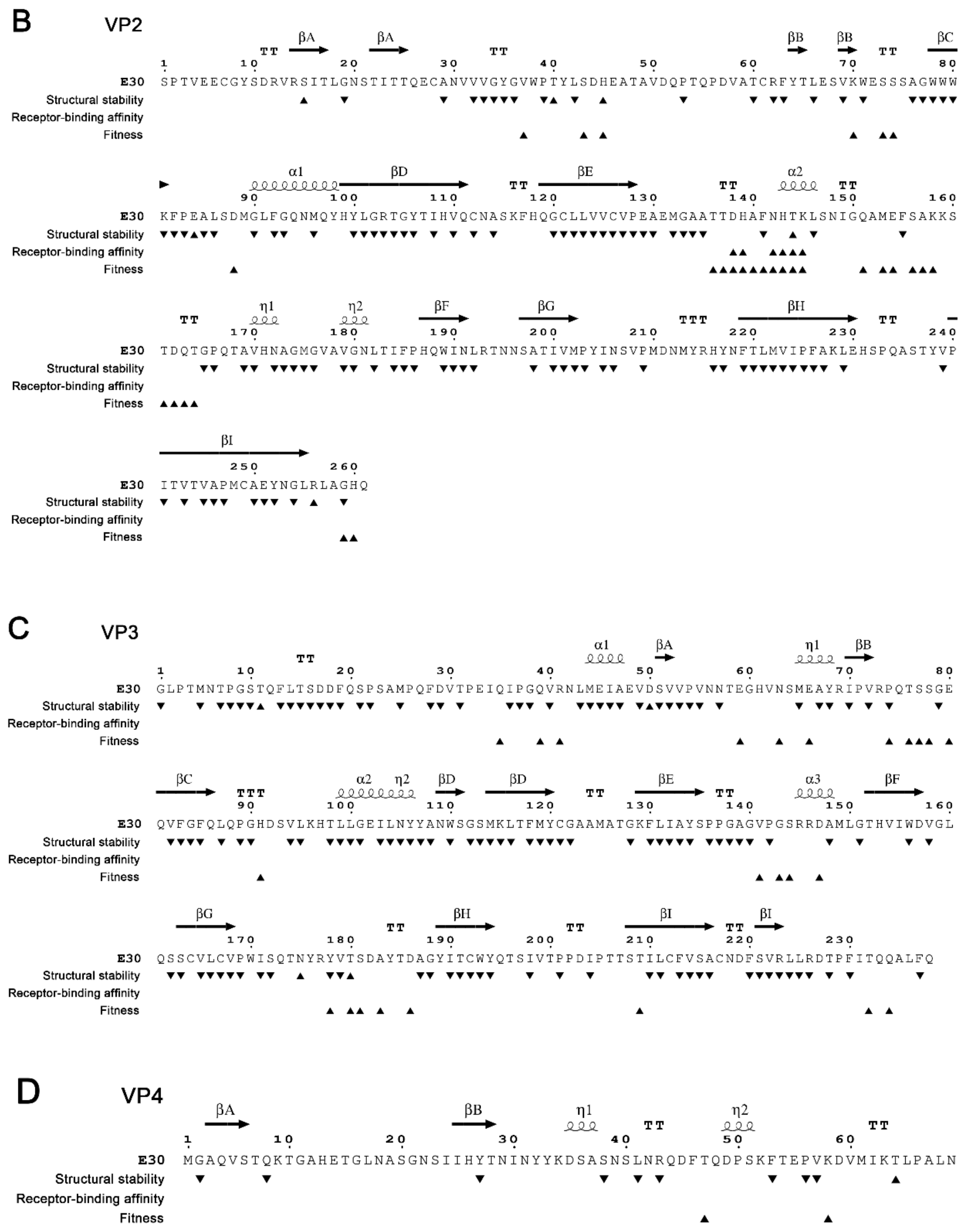

Figure S13. Sequence alignment of risk sites for E30.

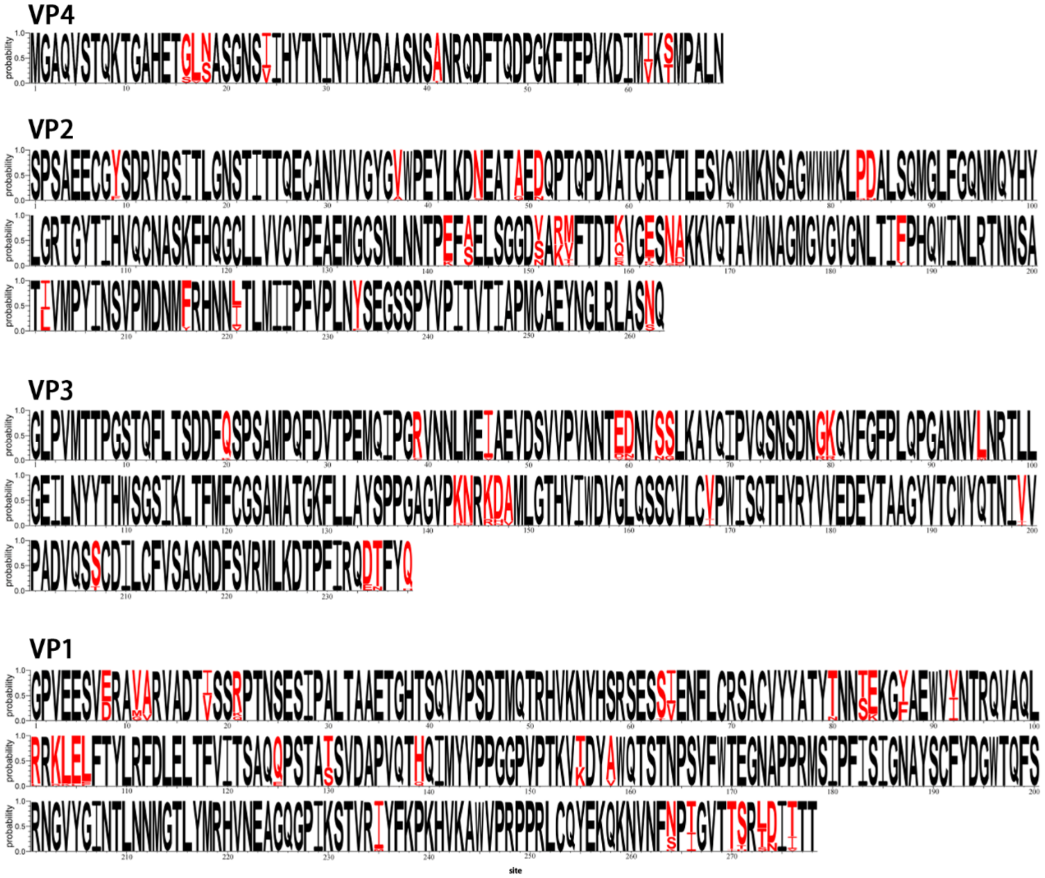

Figure S14. The mutation profile of CVB1.

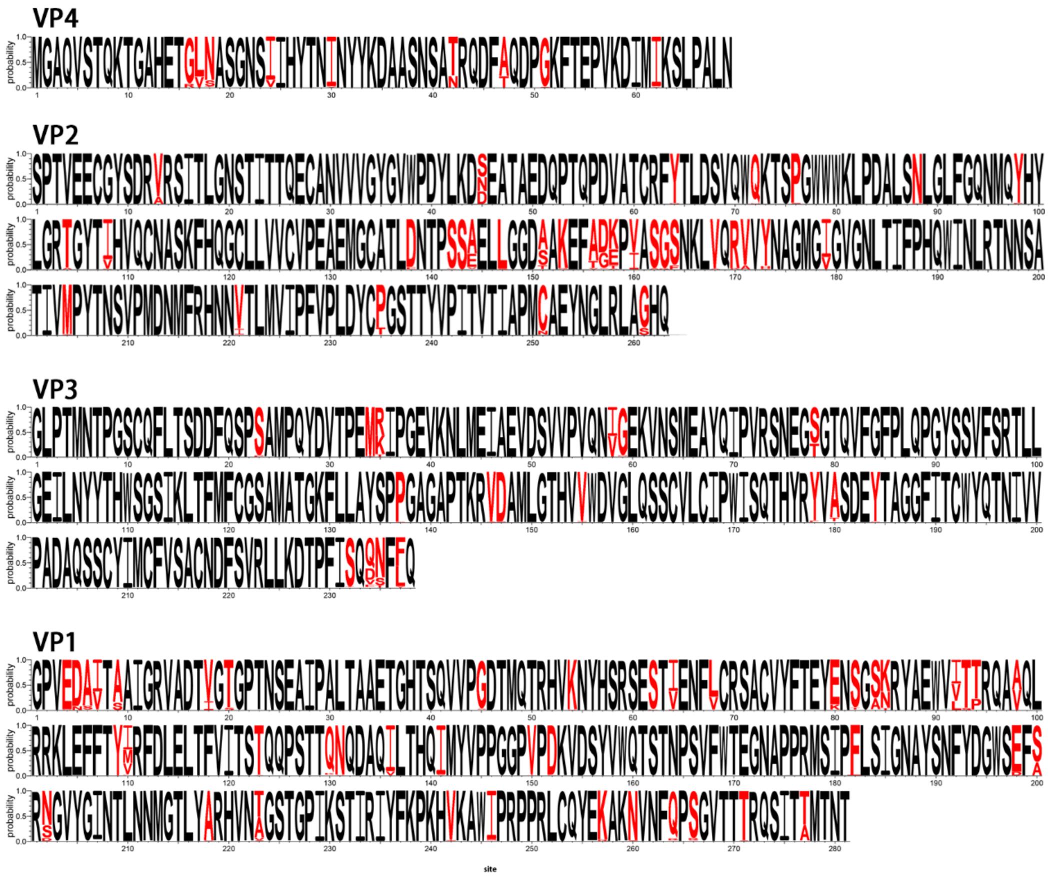

Figure S15. The mutation profile of CVB3.

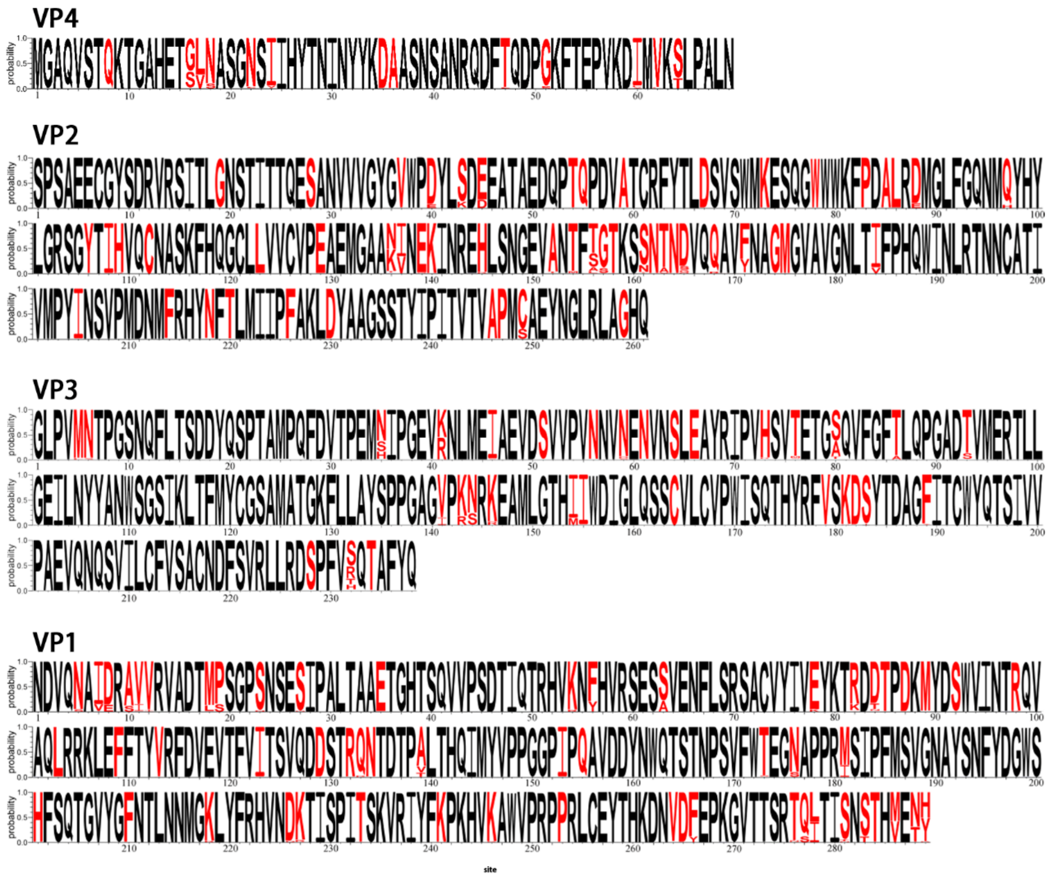

Figure S16. The mutation profile of E6.

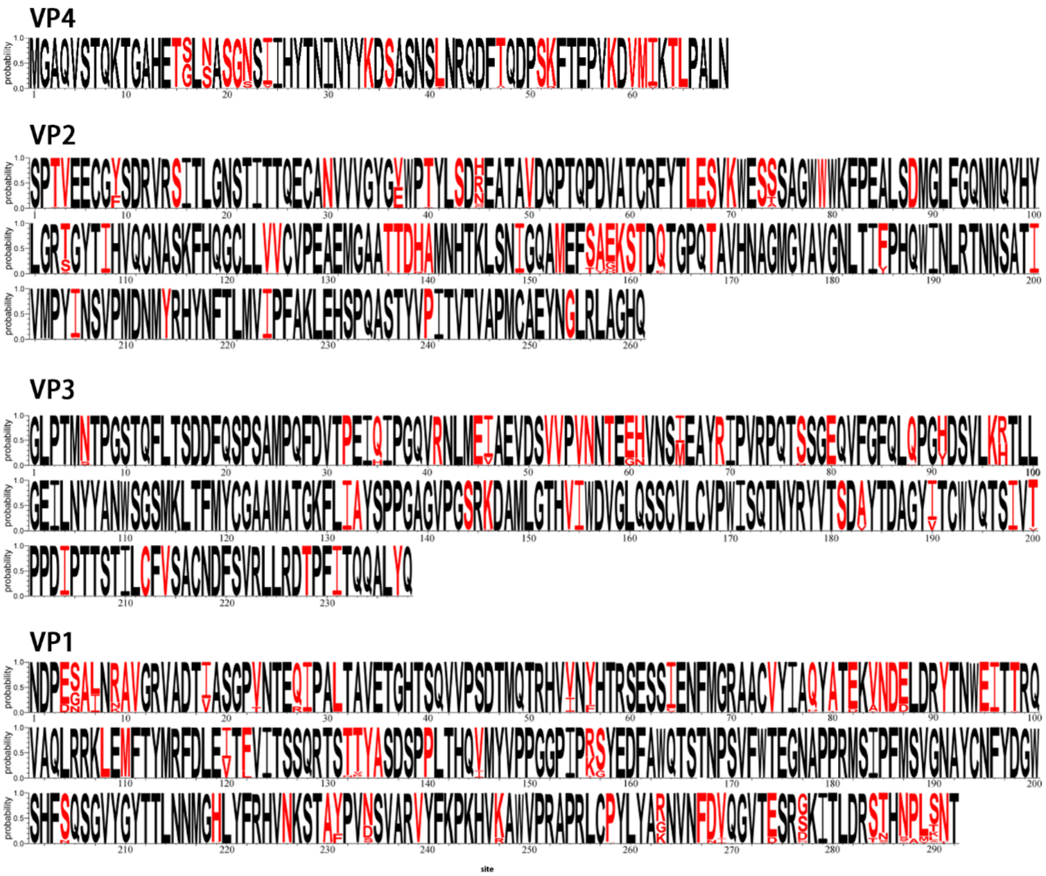

Figure S17. The mutation profile of E30.

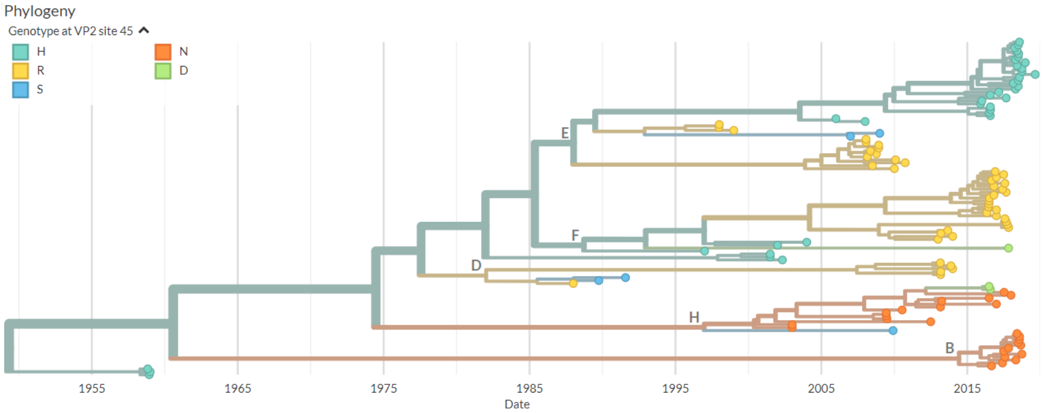

Figure S18. Multiple mutations have occurred at VP2-45, a high-risk site in E30.
